# Home Treatment of Older People with Symptomatic SARS-CoV-2 Infection (COVID-19): A structured Summary of a Study Protocol for a Multi-Arm Multi-Stage (MAMS) Randomized Trial to Evaluate the Efficacy and Tolerability of Several Experimental Treatments to Reduce the Risk of Hospitalisation or Death in outpatients aged 65 years or older (COVERAGE trial)

**DOI:** 10.1186/s13063-020-04619-1

**Published:** 2020-10-13

**Authors:** Alexandre Duvignaud, Edouard Lhomme, Thierry Pistone, Racha Onaisi, Rémi Sitta, Valérie Journot, Duc Nguyen, Nathan Peiffer-Smadja, Antoine Crémer, Stéphane Bouchet, Thomas Darnaud, Delphine Poitrenaud, Lionel Piroth, Christine Binquet, Jean-François Michel, Benjamin Lefèvre, David Lebeaux, Josselin Lebel, Julie Dupouy, Caroline Roussillon, Anne Gimbert, Linda Wittkop, Rodolphe Thiébaut, Joanna Orne-Gliemann, Jean-Philippe Joseph, Laura Richert, Xavier Anglaret, Denis Malvy, the COVERAGE study group

**Affiliations:** 1 CHU Bordeaux, Department of Infectious Diseases and Tropical Medicine, Division of Tropical Medicine and Clinical International Health, F-33000 Bordeaux, France; 2 Inserm U1219, Univ. Bordeaux, IRD, F-33000 Bordeaux, France; 3 Univ. Bordeaux, Inserm, CHU Bordeaux, CIC 1401, EUCLID/F-CRIN Clinical Trials Platform, F-33000 Bordeaux, France; 4 Univ. Bordeaux, Department of Public Health, Inserm Bordeaux Population Health Research Centre, Inria SISTM, F-33000 Bordeaux, France; 5 CHU Bordeaux, Pôle de Santé Publique, F-33000 Bordeaux, France; 6 Department of General Practice, Univ. Bordeaux, F-33000 Bordeaux, France; 7 CHU Bichat Claude Bernard, Department of Infectious Diseases and Tropical Medicine, APHP, F-75000 Paris, France; 8 Université de Paris, IAME, INSERM, F-75018 Paris, France; 9 National Institute for Health Research Health Protection Research Unit in Healthcare Associated Infections and Antimicrobial Resistance, Imperial College London, London, UK; 10 Department of Cardiology – Hypertension, CHU Bordeaux, F-33000 Bordeaux, France; 11 Inserm U1219, Univ. Bordeaux, F-33000 Bordeaux, France; 12 Department of Pharmacology and Toxicology, CHU Bordeaux, F-33000 Bordeaux, France; 13 Centre Hospitalier de Bastia, Service de Chirurgie Spécialisée & Unité de Recherche Clinique, F-20200 Bastia, France; 14 Department of Infectious Diseases and Tropical Medicine, Centre Hospitalier d’Ajaccio, F-20090 Ajaccio, France; 15 Department of Infectious Diseases and Tropical Medicine, CHU Dijon, F-21079 Dijon, France; 16 Inserm U1432, Univ. Bourgogne, F-21000 Dijon, France; 17 Inserm, CHU Dijon, CIC-EC 1432, F-21000 Dijon, France; 18 Centre Medical de Steinsel, Steinsel, Luxembourg; 19 Formation Spécialisée en Medecine Generale (FSMG), Université de Luxembourg, Luxembourg City, Luxembourg; 20 Department of Infectious Diseases and Tropical Medicine, CHU Nancy, F-54000 Nancy, France; 21 Department of Infectious Diseases and Tropical Medicine, Hôpital Européen Georges Pompidou, APHP, F-75000 Paris, France; 22 Department of General Practice, Université de Paris, F-75018 Paris, France; 23 UMR 1137, INSERM, IAME, F-75018 Paris, France; 24 MSPU Pins Justaret, F-31860 Pins Justaret, France; 25 Department of General Practice, Univ. Paul Sabatier, F-31000 Toulouse, France; 26 UMR 1027 Inserm Univ. Paul Sabatier, F-31000 Toulouse, France; 27 CHU Toulouse, CIC, F-31000 Toulouse, France; 28 Clinical Research and Innovation Department, Safety and vigilance, CHU Bordeaux, F-33000 Bordeaux, France; 29 Clinical Research and Innovation Department, CHU Bordeaux, F-33000 Bordeaux, France; 30 Univ. Bordeaux, Department of Public Health, Inserm Bordeaux Population Health Research Centre, MORPH3EUS, F-33000 Bordeaux, France; 31 Department of General Practice, CIC 1401, Univ. Bordeaux, F-33000 Bordeaux, France

## Abstract

**Objectives:**

To assess the efficacy of several repurposed drugs to prevent hospitalisation or death in patients aged 65 or more with recent symptomatic SARS-CoV-2 infection (COVID-19) and no criteria for hospitalisation.

**Trial design:**

Phase III, multi-arm (5) and multi-stage (MAMS), randomized, open-label controlled superiority trial.

Participants will be randomly allocated 1:1:1:1:1 to the following strategies:

- Arm 1: Control arm
- Arms 2 to 5: Experimental treatment arms

Planned interim analyses will be conducted at regular intervals. Their results will be reviewed by an Independent Data and Safety Monitoring Board. Experimental arms may be terminated for futility, efficacy or toxicity before the end of the trial. New experimental arms may be added if new evidence suggests that other treatments should be tested.

A feasibility and acceptability substudy as well as an immunological substudy will be conducted alongside the trial.

**Participants:**

Inclusion criteria are: 65-year-old or more; Positive test for SARS-CoV-2 on a nasopharyngeal swab; Symptoms onset within 3 days before diagnosis; No hospitalisation criteria; Signed informed consent; Health insurance.

Exclusion criteria are: Inability to make an informed decision to participate (*e.g*.: dementia, guardianship); Rockwood Clinical Frailty Scale ≥7; Long QT syndrome; QTc interval > 500 ms; Heart rate <50/min; Kalaemia >5.5 mmol/L or <3.5 mmol/L; Ongoing treatment with piperaquine, halofantrine, dasatinib, nilotinib, hydroxyzine, domperidone, citalopram, escitalopram, potent inhibitors or inducers of cytochrome P450 CYP3A4 isoenzyme, repaglinide, azathioprine, 6-mercaptopurine, theophylline, pyrazinamide, warfarin; Known hypersensitivity to any of the trial drugs or to chloroquine and other 4-aminoquinolines, amodiaquine, mefloquine, glafenine, floctafenine, antrafenine, ARB; Hepatic porphyria; Liver failure (Child-Pugh stage ≥B); Stage 4 or 5 chronic kidney disease (GFR <30 mL/min/1.73 m²); Dialysis; Hypersentivity to lactose; Lactase deficiency; Abnormalities in galactose metabolism; Malabsorption syndrome; Glucose-6-phosphate dehydrogenase deficiency; Symptomatic hyperuricemia; Ileus; Colitis; Enterocolitis; Chronic hepatitis B virus disease.

The trial is being conducted in France in the Bordeaux, Corse, Dijon, Nancy, Paris and Toulouse areas as well as in the Grand Duchy of Luxembourg. Participants are recruited either at home, nursing homes, general practices, primary care centres or hospital outpatient consultations.

**Intervention and comparator:**

The four experimental treatments planned in protocol version 1.2 (April 8^th^, 2020) are: (1) Hydroxychloroquine 200 mg, 2 tablets *BID* on day 0, 2 tablets *QD* from day 1 to 9; (2) Imatinib 400 mg, 1 tablet *QD* from day 0 to 9; (3) Favipiravir 200 mg, 12 tablets *BID* on day 0, 6 tablets *BID* from day 1 to 9; (4) Telmisartan 20 mg, 1 tablet *QD* from day 0 to 9.

The comparator is a complex of vitamins and trace elements (AZINC Forme et Vitalité®), 1 capsule *BID* for 10 days, for which there is no reason to believe that they are active on the virus.

In protocol version 1.2 (April 8th, 2020): People in the control arm will receive a combination of vitamins and trace elements; people in the experimental arms will receive hydroxychloroquine, or favipiravir, or imatinib, or telmisartan.

**Main outcome:**

The primary outcome is the proportion of participants with an incidence of hospitalisation and/or death between inclusion and day 14 in each arm.

**Randomisation:**

Participants are randomized in a 1:1:1:1:1 ratio to each arm using a web-based randomisation tool. Participants not treated with an ARB or ACEI prior to enrolment are randomized to receive the comparator or one of the four experimental drugs. Participants already treated with an ARB or ACEI are randomized to receive the comparator or one of the experimental drugs except telmisartan (*i.e*.: hydroxychloroquine, imatinib, or favipiravir). Randomisation is stratified on ACEI or ARBs treatment at inclusion and on the type of residence (personal home *vs*. nursing home).

**Blinding (masking):**

This is an open-label trial. Participants, caregivers, investigators and statisticians are not blinded to group assignment.

**Numbers to be randomised (sample size):**

A total of 1057 participants will be enrolled if all arms are maintained until the final analysis and no additional arm is added.

Three successive futility interim analyses are planned, when the number of participants reaches 30, 60 and 102 in the control arm. Two efficacy analyses (interim n°3 and final) will be performed successively.

**Trial Status:**

This describes the Version 1.2 (April 8^th^, 2020) of the COVERAGE protocol that was approved by the French regulatory authority and ethics committee. The trial was opened for enrolment on April 15^th^, 2020 in the Nouvelle Aquitaine region (South-West France). Given the current decline of the COVID-19 pandemic in France and its unforeseeable dynamic in the coming months, new trial sites in 5 other French regions and in Luxembourg are currently being opened. A revised version of the protocol was submitted to the regulatory authority and ethics committee on June 15^th^, 2020. It contains the following amendments: (i) Inclusion criteria: age ≥65 replaced by age ≥60; time since first symptoms <3 days replaced by time since first symptoms <5 days; (ii) Withdrawal of the hydroxychloroquine arm (due to external data); (iii) increase in the number of trial sites.

**Trial registration:**

The trial was registered on Clinical Trials.gov on April 22^nd^, 2020 (Identifier: NCT04356495): and on EudraCT on April 10^th^, 2020 (Identifier: 2020-001435-27).

**Full protocol:**

The full protocol is attached as an additional file, accessible from the Trials website (Additional file 1). In the interest of expediting dissemination of this material, the familiar formatting has been eliminated; this Letter serves as a summary of the key elements of the full protocol. The study protocol has been reported in accordance with the Standard Protocol Items: Recommendations for Clinical Interventional Trials (SPIRIT) guidelines (Additional file 2).

## Supplementary information

**Additional file 1.** COVERAGE trial protocol version 1.2 (April 8th 2020).

**Additional file 2.** SPIRIT 2013 Checklist: Recommended items to address in a clinical trial protocol and related documents.

**Additional file 3.** List of members of the COVERAGE study group.

## Data Availability

Not applicable.

