# Supplementary material for: Home Treatment of Older People with Symptomatic SARS-CoV-2 Infection (COVID-19): A structured Summary of a Study Protocol for a Multi-Arm Multi-Stage (MAMS) Randomized Trial to Evaluate the Efficacy and Tolerability of Several Experimental Treatments to Reduce the Risk of Hospitalisation or Death in outpatients aged 65 years or older (COVERAGE trial)
Source: Trials. 2020 Oct 13;21:846. doi: 10.1186/s13063-020-04619-1 (PMC7552584; doi:10.1186/s13063-020-04619-1)
Supplement: Supplementary file 1 — Additional file 1. COVERAGE trial protocol version 1.2 (April 8th 2020). [file 13063_2020_4619_MOESM1_ESM.pdf]

BORDEAUX UNIVERSITY HOSPITAL

Home Treatment of Elderly People with Symptomatic SARS CoV-2 Infection (COVID-19): A Multi-Arm Multi-Stage Randomized Trial (MAMS) to Evaluate the Efficacy and Tolerability of Several Experimental Treatments to Reduce the Risk of Hospitalization or Death

COVERAGE trial

Sponsor Code: CHUBX 2020/12

RESEARCH PROTOCOL

HUMAN-INTERVENTIONAL

(Category 1 research)

Version n°1.2 April 8th, 2020

EudraCT Number: 2020-001435-27

Clinicaltrials.gov : NCT04356495

In the emergency context, this intervention research received seed funding from the French National Research Agency (ANR).

Sponsor:
Centre Hospitalier Universitaire de Bordeaux, 12 rue Dubernat, 33400 Talence, France

Coordinating Investigator :
Pr Denis MALVY, Centre Hospitalier Universitaire de Bordeaux, 12 rue Dubernat, 33400 Talence, FRANCE

Clinical trial units:

EUCLID Clinical Trial Plateform (F-CRIN), CIC-1401, ISPED, University of Bordeaux, 146 rue Léo Saignat, 33076 Bordeaux Cedex, FRANCE

MEREVA, IDLIC team, Centre Inserm 1219, University of Bordeaux, 146 rue Léo Saignat, 33076 Bordeaux Cedex.

Clinical Research Safety and Vigilance Unit:
Direction of Clinical Research and Innovation, 12 rue Dubernat, 33404 Talence Cedex, France
Tel: 05 57 82 16 26 - Fax: 05 57 82 12 62
vigilance.essais-cliniques@chu-bordeaux.fr

PROTOCOL VERSIONS

Version #	Date	Amendment #	Reasons for the update
1.0	31/03/2020		
1.1	04/04/2020	-	Response to the ethic commitee's comments
1.2	08/04/2020	-	Response to ANSM comments

PROTOCOL SIGNATURE PAGE

Home Treatment of Elderly People with Symptomatic SARS CoV-2 Infection (COVID-19): A Multi-Arm Multi-Stage Randomized Trial (MAMS) to Evaluate the Efficacy and Tolerability of Several Experimental Treatments to Reduce the Risk of Hospitalization or Death

Sponsor Code: CHUBX 2020/12

Sponsor

Bordeaux University Hospital
12 Dubernat Street
33400 Talence
FRANCE

in Bordeaux, on: 08/04/2020

The Director General of the Bordeaux University Hospital
Y. BUBIEN
And by delegation, the Director of Clinical Research and Innovation,
J. BELCASTRO

Coordinating Investigator

Prof. Denis MALVY
Bordeaux University Hospital Center,
12 Dubernat Street,
33,400 Talent
FRANCE
Email: denis.malvy@chu-bordeaux.fr

in Bordeaux, on: 08/04/2020

Professor D. MALVY

MAIN CORRESPONDENTS

**Coordinating Investigator**<br>Prof. Denis MALVY<br>Bordeaux University Hospital Center,<br>12 Dubernat Street,<br>33,400 Talent<br>FRANCE<br>Email: denis.malvy@chu-bordeaux.fr<br><br>**Coordinating Pharmacist**<br>Dr Sarah DJABAROUTI<br>Bordeaux University Hospital<br>12 Dubernat Street,<br>33,400 Talent<br>FRANCE<br>sarah.djabarouti@chu-bordeaux.fr<br><br>**Virology laboratory**<br>Pr Marie-Edit Lafon<br>CHU de Bordeaux<br>12 rue Dubernat,<br>33 400 Talence<br>Tel : 05 56 79 55 10<br>Email : marie-edith.lafon@chu-bordeaux.fr	**Sponsor**<br>Bordeaux University Hospital<br>12 rue Dubernat<br>33,400 Talent<br>FRANCE<br><br>**Responsible for research at the sponsor level**<br>Jonathan BELCASTRO - Director of Clinical Research and Innovation<br>Dr Anne GIMBERT - Head of "Internal promotion".<br>Tel : 05 57 82 08 34 - Fax : 05 56 79 49 26<br>Email: anne.gimbert@chu-bordeaux.fr<br><br>**Clinical Research Safety and Vigilance Unit**<br>Dr. Caroline ROUSSILLON (Medical Evaluator),<br>Clinical Research and Innovation Directorate<br>12 Dubernat Street<br>33404 Talence Cedex<br>Tel: 05 57 82 16 26 - Fax: 05 57 82 12 62<br>vigilance.essais-cliniques@chu-bordeaux.fr<br><br>**Clinical trial units**<br>EUCLID Clinical Trial Plateform (F-CRIN)<br>Dr Laura RICHERT<br>Laura.Richert@u-bordeaux.Fr<br><br>MEREVA, IDLIC team, Inserm 1219 centre<br>Dr Xavier ANGLARET<br>Xavier.Anglaret@u-bordeaux.fr

ABBREVIATIONS

AE	Adverse Event
ALT	Alanine aminotransferase
ANRS	French Agency for Research on AIDS and Viral Hepatitis
AR	Adverse Reaction
AST	Aspartate aminotransferase
AUC	Area Under a Curve
BIS	"*Bis in die*" (twice a day)
CHU	University Hospital Center
CRA	Clinical Research Assistant
CRF	Case Report Form
CTU	Clinical Trial Unit
D	Day
DNA	Deoxyribonucleic acid
DSMB	Data & Safety Monitoring Board
eTMF	electronic Trial Master File
ICH	International Council on Harmonization
INSERM	National Institute of Health and Medical Research
ITT	Intent-to-treat analysis
PP	Per protocol analysis
QD	*“Quaque die”* (once a day)
RNA	Ribonucleic acid
SAB	Scientific Advisory Board
SAE	Serious Adverse Event
SAR	Serious Adverse Reaction (Serious Adverse Reaction)
SDPM	See Details Procedures Manual
SOP	Standard Operating Procedure
SPC	Summary of product characteristics
SUSAR	Suspected Unexpected Serious Adverse Reaction
WHO	World Health Organization

SUMMARY

**SPONSOR**	CHU de Bordeaux
**COORDINATING INVESTIGATOR**	Denis MALVY
**TITLE**	Home treatment of elderly patients with symptomatic SARS-CoV-2 infection (COVID-19) : a multiarm, multi-stage (MAMS) randomized trial to assess the efficacy and safety of several experimental treatments to reduce the risk of hospitalization or death (COVERAGE trial)
**BACKGROUND**	In adults aged 65 years or older with COVID-19 disease without hospitalization criteria, early treatment could prevent hospitalization or death. No treatment is currently validated for this indication.
**MAIN OBJECTIVE**	To estimate the efficacy of several specific experimental treatments, compared to standard care, to prevent hospitalization or death at D14 in adults over 65 years of age, with documented SARS-CoV-2 infection, with symptoms lasting less than 72 hours and not meeting any hospitalization criteria.
**BENEFITS FOR THE PARTICIPANTS**	Daily follow-up by a dedicated team.
**DESIGN**	Phase III multi-centre, open-label, randomized controlled superiority multi-arm multi-stage, (MAMS) trial
**INCLUSION CRITERIA**	– Positive SARS-CoV-2 test on nasopharyngeal swab<br>– Onset of symptoms < 72 hours prior to nasopharyngeal swabbing<br>– Age ≥ 65 years old<br>– Valid, ambulatory person, fully capable of understanding the challenges of the trial<br>– No hospitalization criteria according to current recommendations<br>– Signed informed consent<br>– Covered by Health Insurance
**EXCLUSION CRITERIA**	– Inability to make a decision to participate (dementia, guardianship)<br>– Long QT syndrome, or QTc space > 500 ms<br>– Presence of a pace maker<br>– Heart rate <50 / min<br>– Hyperkalaemia > 5.5 mmol/L or hypokalaemia < 3.5 mmol/L<br>– Treatment with piperaquine, halofantrine, dasatinib, nilotinib, citalopram, escitalopram, hydroxyzine, domperidone, potent inhibitors of cytochrome P450 CYP3A4 isoenzyme, potent inducers of cytochrome P450 CYP3A4 isoenzyme repaglinide, azathioprine, 6-mercaptopurine, theophylline, pyrazinamide, warfarin<br>– Hypersensitivity to any of the test drugs or chloroquine and other 4-aminoquinolines, amodiaquine, mefloquine, glafenine, floctafenine, antrafenine, ARB or sartan.<br>– Hepatic porphyria, known obstructive hepato-biliary disease, liver failure (stage ≥ Child-Pugh B), stage 4 or 5 chronic kidney disease (DFG <30 mL/min/1.73 m²) or person on dialysis, retinopathy, lactose hypersensitivity, abnormalities in galactose metabolism, lactase deficiency, malabsorption syndrome, known glucose-6-phosphate dehydrogenase deficiency, symptomatic gout/hyperuricemia, ileus, colitis or enterocolitis, chronic infection with the hepatitis B virus
**TREATMENTS**	– AZINC Forme et Vitalité®: (comparator ; vitamins + trace elements complex)<br>– Plaquenil® (Hydroxychloroquine)

	– Avigan® (Favipiravir)<br>– Imatinib TEVA®<br>– Micardis® (Telmisartan)<br>Treatments will be given for 10 days. People who were on angiotensin conversion enzyme inhibitor (ACEI) or ARB treatment before inclusion will not be randomized to the Telmisartan arm
**MAIN OUTCOME**	Proportion of participants with an occurrence of hospitalization and/or death between D0 and D14 in each arm
**NUMBER OF CENTERS**	The study takes place on an outpatient basis with follow-up at home and by telephone.
**DURATION**	Inclusion period: 3 months<br>Participation duration: 28 days.
**ANALYSIS**	This is a Phase III trial. Sequential analyses will be performed for the primary endpoint at D14, with the following objectives:<br>- Intermediate analysis #1 (30 included patients per arm): futility<br>- Intermediate analysis n°2 (60 patients included per arm): futility<br>- Intermediate analysis n°3 (102 patients included per arm): futility and early efficacy<br>- Final analysis: efficacy
**NUMBER OF PARTICIPANTS**	- 169 participants in each of the 5 arms in people who were not on basic therapy with IEC or ARA2 before inclusion, i.e. 845 patients in total if the 5 arms are maintained until the end.<br>- 53 participants in each of the 4 arms (excluding telmisartan) in people who were on treatment with IEC or ARA2 before inclusion, i.e. 212 more patients if the 4 arms are maintained until the end.<br>The final number will depend on the number of arms that will continue after the intermediate analyzes and could not excess 1057 participants
**EXPECTED BENEFITS**	Treatments to prevent complications of COVID-19 in the elderly. French hospitals are overwhelmed with new COVID-19 patients and it is expected that beds in the intensive care units will run out in the near future. Finding an effective treatment that can be administered at home to avoid hospitalization and transfer to intensive care is of critical importance.
**SIMULTANEOUS PARTICIPATION IN ANOTHER RESEARCH AND EXCLUSION PERIOD**	The inclusion and simultaneous participation in any other interventional study is not authorized in order not to interfere with the results of this research.<br>No exclusion period is necessary following this research.

Table of content

1 CONTEXT AND JUSTIFICATION 11
1.1 Context 11
1.2 Justification 11
1.3 Data on the effectiveness of drugs against SARS-CoV-2 11
1.3.1 Protease inhibitors 12
1.3.2 Chloroquine and hydroxychloroquine 12
1.3.3 Imatinib 12
1.3.4 Favipiravir 13
1.3.5 Angiotensin receptor blockers (ARB) 13
1.4 Methodological justification 13
1.4.1 Choice of study model 13
1.4.2 Choice of main judgement criterion 14
1.4.3 Choice of treatments 14
1.4.4 Choice of studied population 14
2 PRIOR PROJECTS OF THE TEAM 15
3 OBJECTIVES 15
3.1 Main objective 15
3.2 Secondary objectives 15
4 Trial model 16
4.1 Study phase and classification 16
4.2 Groups of treatments 16
4.2.1 Choice of treatments at the time of drafting the protocol 16
4.2.2 Evolution in the choice of treatments 16
4.3 Judgement criteria 16
4.3.1 Main criterion 16
4.3.2 Secondary criteria 16
4.4 Randomisation 17
5 METHODS 17
5.1 Eligibility criteria 17
5.1.1 Inclusion criteria 17
5.1.2 Non-inclusion criteria 17
5.2 Trial treatments 18
5.2.1 Vitamins 18
5.2.2 Hydroxychloroquine 18
5.2.3 Imatinib 18
5.2.4 Favipiravir 18
5.2.5 Telmisartan 18
5.2.6 Concomitant drugs 19
5.2.7 Experimental drugs circuit 19
5.3 Progress of the trial 19
5.3.1 Overview 19
5.3.2 Period before inclusion 20
5.3.3 Inclusion (Day 0) 20
5.3.4 Remote monitoring of the ECG and consideration of the blood electrolytes for inclusion 21
5.3.5 Remote visits (Days 1, 3, 5, 9 and 12) 21
5.3.6 Face-to-face visits (Day 7 and Day 14) 21
5.3.7 Out of phase contacts during the trial 21

5.3.8 Visit at the end of the trial (Day 28 or later) ... 21
5.3.9 Changes to scheduled visits ... 22
5.3.10 Biological examinations ... 22
5.4 Management of adverse events and new findings ... 22
5.4.1 Definitions ... 22
5.4.2 Description of expected adverse events ... 23
5.4.3 Conduct to be maintained by the researcher in the case of adverse event or new finding ... 23
5.4.4 Declaration by the sponsor of Suspected Unexpected Serious Adverse Reactions and new findings. ... 24
5.4.5 Annual safety report ... 25
5.5 Safety of staff ... 25
6 Statistical analysis ... 25
6.1 Principles ... 26
6.2 Sample Size ... 27
6.2.1 Participants not taking ACEI or ARB before inclusion ... 27
6.2.2 Participants taking ACEI or ARB before inclusion ... 27
6.2.3 Overall sample size ... 28
6.3 Analysis plan ... 28
6.3.1 General information on the statistical techniques used ... 28
6.3.2 Accrual and follow-up ... 28
6.3.3 Baseline characteristics ... 28
6.3.4 Primary outcome for participants not taking ACEI or ARB before inclusion ... 28
6.3.5 Primary outcome for participants taking ACEI or ARB before inclusion ... 29
6.3.6 Secondary endpoints ... 29
6.3.7 Safety analysis ... 29
7 Governance ... 29
7.1 Sponsor ... 29
7.2 Trial team ... 29
8 Monitoring ... 30
8.1 Scientific Advisory Board ... 30
8.2 Data & Safety Monitoring Board ... 31
9 Sub-studies ... 31
9.1 Immunological sub-study ... 31
9.1.1 Justification ... 31
9.1.2 Methods ... 31
9.1.3 Laboratory ... 32
9.1.4 Scale of the study ... 32
9.1.5 Statistical analysis ... 32
9.2 Feasibility and take-up sub-study ... 33
9.2.1 Justification ... 33
9.2.2 Methods ... 33
9.3 Data management ... 33
9.3.1 Data management software ... 33
9.3.2 Source data ... 33
9.3.3 Data security ... 34
9.3.4 Entry and control of data ... 34
9.3.5 Control of AE/SAE databases ... 34
9.3.6 Setting the database ... 34
9.3.7 Archiving of the database ... 34
9.3.8 Quality management ... 34
9.3.9 Confidentiality and security of participants' data ... 34
9.4 Monitoring ... 34

9.4.1 Clinical Trial Unit (CTU) 34
9.5 Differences and deviations to the protocol 35
9.5.1 Essential documents 35
9.5.2 Audits, file inspections 35
10 ETHICAL ASPECTS 35
1.1.1 ETHICAL AND REGULATORY CONSIDERATIONS 35
10.1 Protocol modifications 36
10.2 Confidentiality 36
10.3 Insurance and care of participants 36
11 END OF THE STUDY 36
11.1 Publication of results 37
11.2 Impact 37
11.3 Archiving of documents 37
12 DATA TRANSFER 37
13 BIBLIOGRAPHY 39

1 CONTEXT AND JUSTIFICATION

1.1 Context

This protocol has been written in a context of extreme urgency. The epidemic, which is destabilising the care system of numerous countries and several regions of France, is growing in power in the Nouvelle-Aquitaine region. In a few days, it is possible that the care systems will be overwhelmed in some areas of the country. In this context, home-based treatment of people affected by COVID-19 will become a major part of the response, to provide relief for emergency and hospital services, by avoiding clinical deterioration in people who are most at risk of developing a severe form of the disease, requiring hospitalisation. There is therefore a twin threat to individual and public health level.

The urgent implementation of a home-based screening and follow-up model, allowing clinical monitoring according to the regulatory and ethical requirements of clinical research, raises enormous logistical challenges. The solutions adopted should be adjusted to circumstances, including shortages of Personal Protective Equipment or tests and clogging of the emergency services, screening centres or laboratories. This adjustment will perhaps require rapid organisational decisions in the days before the start of the trial, perhaps even during it. This is the reason why the text of this protocol refers back to the procedures for certain roll-out details, with the note "[SDPM]" {See Details in Standard Operating Procedures]". The drafting and evolution of such procedures shall be monitored in particular: (i) by the Scientific Advisory Board (SAB) and by the independent data and safety monitoring board (DSMB), which meet on a frequent basis, (ii) by the Institutional Review Board (IRB), to authorise implementation.

1.2 Justification

For adults aged 65 years or more affected by the disease COVID-19 at an early phase and without hospitalisation criteria, early treatment could prevent hospitalisation or death. No treatment has currently been validated in this indication (1). The majority of clinical trials for COVID-19 in progress or planned are hospital-centered and enrol patients at a later stage of the disease (2). Optimising treatment at home could reduce the workload in hospitals and improve individual results. This is one of the greatest challenges to be overcome in the weeks to come.

Among risk factors of clinical deterioration in patients with COVID-19, age is described as the most important (3–6). Admission of elderly people suffering from the disease in Intensive Care Units is sometimes limited, justifying the major interest in avoiding the severe forms, which are not necessarily treated in the best way, in the context of an epidemic (7,8).

We are proposing a trial to evaluate the tolerance and efficacy of potential drugs for this indication, while considering the following points:

- Preclinical arguments suggesting an antiviral activity against SARS-CoV-2 already exist for several drugs. Numerous pre-clinical and clinical research projects are in progress on this topic. Ongoing clinical trials must be able to adapt and rapidly change their therapeutic strategies, when new evidence emerges.
- Rapid implementation of a clinical trial for people confined at home poses numerous challenges. Feasibility should be checked, without delaying the evaluation of efficacy.

These points imply the need for a trial comparing the absence of a specific curative treatment (current situation) with experimental curative treatments: (i) starting with available drug candidates, yet allowing consideration of new candidates at any stage; (ii) allowing the interruption of inefficacious treatments (on the basis of futility criteria) or toxic ones.

In response to this situation, we have designed the project as a multi arms and multi stages (MAMS) randomised controlled trial. The trial is to start in Bordeaux and the surrounding area. It could be extended to several European or African partner countries. Discussions have already begun on this with Spain, Italy, Ivory Coast, Senegal and Burkina Faso. In the event of being extended, each country will have its own protocol, yet with a common design, allowing common analyses. The current version of the protocol describes the study, which is being rolled out in Bordeaux and the surrounding area.

1.3 Data on the effectiveness of drugs against SARS-CoV-2

There are available data on the effectiveness of several drugs against SARS-CoV-2 and other coronaviruses. They mainly come from *in vitro* studies and rarely from animal and human studies. Currently, there is no data from randomised trials showing the clinical benefits of using one of these drugs. Many are in progress or planned, but of the 115 ongoing

therapeutic trials targeting SARS-CoV-2 infection registered up to March 7th 2020 on clinicaltrials.gov, WHO and the Chinese registers of clinical trials, we have found no trial specifically targeting ambulatory patients presenting at increased risk of hospitalization or death (2).

1.3.1 Protease inhibitors

Lopinavir/ritonavir (LPV/r) is a fixed-dose combination used to treat HIV infection. It has shown to be effective *in vitro* against SARS-CoV-2 in several studies and seems to be effective against MERS-CoV in animal studies (9). Lopinavir and ritonavir link up *in vitro* at the active site of the SARS-CoV protease (10) and initial virtual anchoring studies have confirmed that the spatial structure of the linkage site of LPV/r is conserved between SARS-CoV and the SARS-CoV-2 (11). Within a cohort of 111 patients infected with SARS-CoV, use of LPV/r has been associated with better clinical results (12). Use of this agent in treating COVID-19 has been described in case series of patients infected with SARS CoV-2 in Singapore, with variable clinical results (13–20). This data has led to the registration of more than 15 clinical trials of LPV/r for COVID-19 in the clinical trials register of USA and China. WHO shortlisted LPV/r as a promising candidate for the treatment of COVID-19 (16). LPV/r has been included as a recommended treatment for COVID-19 in dsome national guidelines, including in China and France (17,18). Even if a randomised trial held in China has not demonstrated the superiority of LPV/r as compared to standard of care (19), this drug should continue to be evaluated. The problem is that ritonavir interacts with numerous drugs, which might be taken by elderly people and quite frequently causes secondary digestive effects, especially diarrhoea, which could, in association with COVID-19, lead to severe dehydration (20). We have therefore not retained this option.

We intended to replace it with another protease inhibitor, darunavir/cobicistat, suggested as a potential candidate for treating COVID-19 (21). A molecular modelling study suggests that darunavir/cobicistat could attach itself to the papain domain of SARS-CoV-2 viral protease, bringing about small changes in the conformation of the protease (22). Use of darunavir/cobicistat for COVID-19 has been reported in a pre-publication by Chinese authors (23). Four clinical trials are being prepared. It is also mentioned as a second line option in the treatment of COVID-19 patients in Chinese recommendations (24). Johnson & Johnson, however, has recently highlighted the absence of supportive evidence for the use of darunavir/cobicistat at this stage (25). Even though better tolerated than LPV/r, nevertheless, we have no longer retained this trial, for fear of risking potential drug-drug interactions with ritonavir or cobicistat among elderly.

1.3.2 Chloroquine and hydroxychloroquine

Even if the efficacy of chloroquine on a certain number of viruses has been described *in vitro* (26,27), it has not demonstrated any clinical benefit in treating influenza, dengue or chikungunya (28–30). Since the start of the COVID-19 pandemic, several publications have reported some efficacy of chloroquine against COVID-19 and mentioned clinical efficacy (31–37). However, published studies to date are either non-comparative or of an insufficient sample size. Chloroquine having previous usage, being easy to administer and well tolerated, it is logical, in the absence of any conclusive result, to evaluate it in an outpatient treatment trial. The mechanism action of chloroquine and hydroxychloroquine being similar (36) and the pharmacokinetic data showing that hydroxychloroquine was more powerful on cells infected with SARS-CoV-2 than the chloroquine phosphate (37) it is also logical to use hydroxychloroquine rather than chloroquine in such trials, especially as pharmacokinetic models predict that hydroxychloroquine could have a better diffusion in pulmonary fluids (37) and as it is better tolerated.

In the light of such data, on March 7th 2020, 18 clinical trials evaluating hydroxychloroquine or chloroquine have been registered on clinicaltrials.gov. The dose for COVID-19 to be evaluated has been a matter of debate. In a recent, single-arm trial in France, the daily hydroxychloroquine dose was 600mg. Chinese guidelines on COVID-19 currently recommend a dose of 400mg twice a day for 2 days, followed by 200mg twice a day over 4 days. Italian guidelines also use a loading dose of 400mg per day over 2 days, followed by a maintenance dose of 200mg over 8 days. Recent pharmacokinetic modelling suggests that a loading dose of 400mg twice a day for the first day, followed by 200mg twice daily over 5 days should be sufficient to allow the attainment and maintenance of active therapeutic concentrations for SARS-CoV-2 (37). In our trial, we have chosen to use the dose also retained for the DisCovEry trial (NCT04315948).

1.3.3 Imatinib

Imatinib, an Abelson kinase inhibitor (Abl), targets the Abelson tyrosine-protein kinase 2 and has shown *in vitro* effectiveness on a certain number of viruses, among which HIV (38), vaccine (39), coxsackievirus (40) and Ebola virus (41,42). Abl kinase inhibitors block fusion of coronaviruses with the host cell's membrane (43). Replication of SARS-CoV and MERS-CoV is inhibited by imatinib (44,45). This drug has been used for a long time as a chronic treatment in people suffering from chronic myeloid leukaemia, many of them are elderly, and has demonstrated an excellent tolerance (46–48). It is thus an interesting drug to be evaluated in an outpatient trial targeting people over 65 years-old.

1.3.4 Favipiravir

Favipiravir is an antiviral selectively inhibiting the RNA dependent RNA polymerase of the numerous viruses, including influenza, hantaviruses, flaviviruses, arenaviruses, filoviruses, bunyaviruses and enteroviruses (49,50). Its evaluation in the treatment of Ebola virus disease during the epidemic that occurred in West Africa was judged to be non-conclusive in terms of efficacy (51) yet allowed improvements to knowledge of its pharmacokinetics among patients afflicted by a severe viral infection (52). The mechanism of action of favipiravir make it a credible candidate for evaluation in the context of COVID-19. Favipiravir is currently being evaluated in the context of 7 clinical trials for COVID-19. Various brief reports containing non-published data from China and Japan have declared that the preliminary results obtained with favipiravir for COVID-19 patients have been encouraging (53). The preliminary data of a clinical trial suggesting that favipiravir would reduce the duration of viral excretion and improve radiological results in patients infected by SARS-CoV-2 has been communicated in an informal manner by a Shenzhen hospital. Favipiravir at an initial dose of 1,600mg followed by 600mg thrice daily is recommended as an alternative option for patients suffering from COVID-19, who are intolerant of lopinavir/ritonavir among Chinese recommendations (24). On the basis of knowledge of pharmacokinetics of the molecule obtained from the Ebola epidemic (52,54) and of not yet data published regarding the antiviral action of favipiravir in vitro on SARS-CoV2, a regimen using higher doses of favipiravir would have better chances of allowing the attainment of therapeutic concentrations doses among patients suffering from COVID-19. In this trial, we have chosen to use that dose already used during the Ebola epidemic, higher than the dose recommended for influenza in Japan, but already administered under close clinical monitoring in humans.

1.3.5 Angiotensin receptor blockers (ARB)

Finally, the ARB class exhibit promising characteristics for treating COVID-19, not because of its antiviral activity but due to its potentially beneficial effect on the progression of the disease and more particularly on its ability to prevent pulmonary damage. The cellular point of entry for SARS-CoV2 is a membrane enzyme of epithelial cells of the respiratory tract: angiotensin converting enzyme 2 (ACE2) (55). ACE2 is a homologue enzyme of the angiotensin converting enzyme (ACE), which negatively regulates the renin-angiotensin-aldosterone system by converting angiotensin 2 into angiotensin (1-17). It thus has a vasculoprotective effect (56). Animals treated with angiotensin conversion enzyme inhibitors (ACEI) or with ARB have high levels of ACE2 (57). In a mouse model of SARS-CoV infection, a lowering in ACE2 activity has been confirmed. Pulmonary lesions could thus be related to this lowering in ACE2 activity and the excess of angiotensin 2. On the other hand, improvement in the pulmonary condition has been shown in mice treated with an ARB or after injection of recombinant ACE2 (58,59). Thus, a higher level of ACE2 expression among patients under chronic treatment by ARB could protect from developing a severe pulmonary disease when infected by SARS-CoV2 (60). Losartan, telmisartan, olmesartan and other compounds of the ARB class have been widely used since the 1990s for the treatment of arterial hypertension, as well as chronic kidney disease. This class is considered as having few undesirable side effects (61). We make the hypothesis that starting a treatment with telmisartan at a low dose among patients afflicted by COVID-19 at an early stage of their illness could prevent the later deterioration of their respiratory condition, while still being well tolerated.

1.4 Methodological justification

This study shall evaluate the efficacy and safety of several therapeutic strategies against SARS-CoV2 among those aged over 65 not presenting any hospitalisation criterion. We shall conduct a phase III randomised, open-label, controlled superiority clinical trial, with a multi-arm multi-stage (MAMS) framework.

Adults affected by COVID-19 (positive RT-PCR test) with symptoms for fewer than 72 hours at the time of testing and without hospitalisation criterion shall be included in the study and randomised, if they meet the eligibility criteria for inclusion. After randomisation, patients shall receive one of the experimental strategies or a control over 10 days. The main judgement criterion is an evaluation of the efficacy in each treatment group, on the basis of occurrence of hospitalisation or death on Day 14.

1.4.1 Choice of study model

Preclinical and clinical data suggesting the effectiveness of drugs against SARS-CoV-2 are still limited. A trial launched in a context of emergency should be able to integrate those considered suitable under current knowledge, while allowing them to be discarded quickly, if they do not prove immediately promising. Drugs should be able to be included during the course of the trials if new promising evidence arise in the interim.

A multi-arm, multi-stage (MAMS) study would be more efficacious in this context than separated trials with two arms (experimental treatment *vs.* control). This type of trial, offering the opportunity to plan in advance to change one or several aspects of the trial, is particularly suited to the current, highly-evolving, scientific context. MAMS trials allow simultaneous comparison of a certain number of experimental treatments with a single control treatment. This type of

trial provides answers more rapidly and allows the abandonment of arms for futility over planned intermediate analyses or new scientific facts or even to add an arm over the course of the trial.

Three intermediate analyses are planned over the course of the trial, after recruiting 30, 60 and 102 participants per arm. They shall cause the halting of arms, should they prove futile or toxic. At the 3rd intermediate analysis, a preliminary efficacy analysis will allow identification of any treatment, which proves to be efficacious before the end of the trial. This treatment shall then become the standard. A halt on grounds of insufficient safety shall be possible at any point, subject to approval from the DSMB, without the need for a formal statistical criterion.

Multiple statistical tests shall be conducted on grounds of the multi-stage design and the existence of several arms. Type one alpha error shall not be adjusted on grounds of the absence of an objective to extend the indication. The objective is to identify all efficacious drugs and not to identify the best.

Overall, this type of model provides us with a concomitant optimisation of the evaluation of various therapeutic strategies, while adapting itself to the international scientific context and progressively evaluating the most promising strategy or strategies.

1.4.2 Choice of main judgement criterion

The interest of such drugs and their place in the therapeutic strategy of COVID-19 must be judged on a clinical criterion and not only on the evolution of the virological test. We have retained as the main judgement criterion that of "hospitalisation or death". Criteria justifying hospitalisation for COVID-19 shall be standardised and follow current recommendations. This reliable criterion allows an evaluation of the efficacy of early-stage, outpatient treatments.

1.4.3 Choice of treatments

Among people aged over 65 staying at home, treatments must be acknowledged as being well tolerated and not posing drug interaction problems prohibiting their use with very many people.

For the start of the trial, we have therefore retained hydroxychloroquine, favipiravir, telmisartan and imatinib, which seem to present currently *a priori* the best tolerance profile within this population.

We have neither retained lopinavir/ritonavir, entailing too many digestive problems, nor darunavir/ritonavir nor darunavir/cobicistat, which would be better tolerated, but would lead to too many people being discarded on grounds of possible drug interaction problems.

Hydroxychloroquine has been retained as an experimental treatment, not as a benchmark treatment. The researchers for this trial have monitored the controversy, generated as much by the media as the science regarding the efficacy of hydroxychloroquine. Scientifically, there exists limited and contradictory data and no evidence of its clinical efficacy in this indication. At the media level, it is actually possible that the scale of this controversy could render the testing model incomprehensible for the target population. If that were the case, the researchers would seek recommendations of the SAB regarding the necessary changes to the trial model. In such a situation, the hydroxychloroquine arm could become the new control arm (in the absence of convincing evidence of efficacy), thus the relevance of the trial would be reduced. With consideration to the multi-arm model, a difference between hydroxychloroquine and one or the other of the experimental treatments would allow conclusions to be drawn on what would be the meaning of such a difference. Any absence of difference, however, would not be very informative and not allow any conclusions.

Within this population, the issue of multiple drug treatments, with their risks of interaction, cardiac toxicity induced by chloroquine and its derivatives, will be the subject of particular attention. ECG shall be systematic before inclusion, opinion from a cardiologist would be sought in cases of doubt and people included, yet considered as needing to be monitored on this point, would be provided with a self-monitoring ECG device with remote transmission.

1.4.4 Choice of studied population

As for all antiviral treatments, treatment for SARS-CoV-2 would have more chance of being efficacious, if it were administered as quickly as possible after symptoms onset. We have retained as an inclusion criterion "having experienced symptoms for fewer than 72 hours at the time of diagnostic testing".

The main group of the main trial shall be made up of people not taking renin-angiotensin system blockers (ACEI or ARB) before inclusion, who would be randomised between the 5 arms of the study. Calculations for the scale of the study allow for the necessary ability to make comparisons within this group.

People taking renin-angiotensin system blockers (ACEI or ARB) before inclusion represent approximately 20% of patients of the target population for which the study shall be proposed. They cannot be randomised in the Telmisartan arm, but we did not wish to exclude them from the study. They shall therefore be randomised into the 4 other arms and constitute a separate group, which shall be analysed independently. The scale of the study within this group is based on the feasibility of recruitment, using the calculation of the scale of the study of the main group. An extension of the scale

of the study could be made after opinion from the SAB to allow sufficient statistical significance within this group. If the Telmisartan arm of the main group is abandoned in the course of the study on grounds of futility, then people taking renin-angiotensin system blockers (ACEI or ARB) are able to be randomised in the main trial.

2 PRIOR PROJECTS OF THE TEAM

Denis Malvy, coordinating investigator of this project, is the head of the tropical and traveller's diseases clinic at "CHU de Bordeaux". He has implemented and supervised the treatment of suspected or confirmed cases of COVID19 since the start of the epidemic. He is affiliated to the Inserm 1219 Research Centre – IDLIC team at the University of Bordeaux. Within the IDLIC team, he leads the project of "infectious diseases with considerable epidemiological potential", which notably works on viral haemorrhagic fevers. In this regard, he has successfully implemented therapeutic trials and cohort studies in contexts of extreme urgency and logistical challenges during the Ebola epidemic in Guinea in 2014-2015 and the Lassa Fever epidemic in Nigeria in 2018, 2019 and 2020 (51,62,63). The IDLIC team has 25 years of experience in large-scale international therapeutic trials (64) and is in a position to implement a trial on COVID-19 very rapidly. It has available a network of partners across the world, as it is currently coordinating 22 studies occurring in 23 countries (South Africa, Benin, Brazil, Burkina Faso, Cambodia, Cameroon, Ivory Coast, Gambia, Ghana, Guinea, Guinea-Bissau, Madagascar, Mali, Mozambique, Niger, Nigeria, Uganda, Central African Republic, Democratic Republic of Congo, Senegal, Tanzania, Togo and Vietnam). This network can be mobilised very rapidly, as it is currently working on several other communal, multi-site studies. For this study, the IDLIC team will draw from the clinical trials platform, EUCLID/F-CRIN (CIC1401, Bordeaux). This platform is a consortium of Inserm, the CHUs of Bordeaux and Limoges, the University of Bordeaux and the Bergonié Institute of Bordeaux, bringing together expertise in clinical research of the teams from these institutions. EUCLID is one of the clinical trial platforms selected by a call for tenders by F-CRIN (national infrastructure for biology and health, founded in the context of the French future stimulus plan). EUCLID has also been awarded the label "Research Platform for the University of Bordeaux" (2019) and "ECRIN Data Centre" (2015). EUCLID covers all activities required for complex or international academic clinical trials at all stages of clinical development, in collaboration with the sponsor and the coordinating researcher. Since its creation, the platform has undertaken nineteen projects, of which the majority are complex or international trials in the field of infectious diseases.

3 OBJECTIVES

3.1 Main objective

The study's main objective is to appraise the efficacy of several specific experimental treatments, compared with standard of care, to avoid hospitalisation or death on Day 14 among adults older than 65, suffering from symptomatic infection by SARS-CoV-2, presenting symptoms for fewer than 72 hours at the time of testing and having no hospitalisation criterion.

3.2 Secondary objectives

The study's secondary objectives are:

- Appraise the efficacy of several experimental treatments of SARS-CoV-2 infection, compared with standard of care, to prevent death on Day 28
- Appraise the efficacy of several experimental treatments of SARS-CoV-2 infection, compared with standard of care, to prevent hospitalisation on Day 28
- Appraise the efficacy of several experimental treatments of SARS-CoV-2 infection, compared with standard of care, to prevent admission to intensive care unit on Day 28
- Appraise the efficacy of several experimental treatments of SARS-CoV-2 infection to eliminate nasopharyngeal carriage of the virus, compared with standard of care
- Appraise the efficacy of several experimental treatments of SARS-CoV-2 infection, compared with standard of care, to prevent loss of autonomy
- Appraise tolerance of experimental treatments against SARS-CoV-2 infection
- Study those factors associated with clinical deterioration in patients with COVID-19
- Evaluate the feasibility and acceptability of the trial and model of care model implemented.
- Evaluate the relationship between concentrations for each drug and efficacy of each of the treatments

4 Trial model

4.1 Study phase and classification

This study is seamless phase II/III, multicentre, multi-stages and with multiple parallel arms (MAMS), controlled, randomised, superiority clinical trial.

4.2 Groups of treatments

The participants are randomised, in a ratio of 1:1 in each arm, according to the following strategies:

- Arm 1: Treated under standard of care (control arm).
- Arm 2 to X: Experimental treatment (one per arm, X being the number of arms)

4.2.1 Choice of treatments at the time of drafting the protocol

At the time of drafting this protocol:

- The people of the control arm shall receive a food supplement (AZINC Forme et Vitalité®) containing a complex of vitamins and trace elements, for which there is no reason to believe that they are active on the virus.
- Those experimental treatments retained are
  - hydroxychloroquine, favipiravir, imatinib and telmisartan, for people who were not taking renin-angiotensin system blockers (ACEI or ARB) before inclusion.
  - hydroxychloroquine, favipiravir and imatinib, for people who were under treatment by renin-angiotensin system blockers (ACI or ARB) before inclusion.

4.2.2 Evolution in the choice of treatments

All changes to the treatment arms must be approved by the SAB for the trial and be the subject of an amendment, which is submitted to the ethical and regulatory authorities.
This change, which is authorised by the trial model and foreseen within the randomisation procedures, could have two triggers: (i) the scope of the controversy about hydroxychloroquine in the media; and (ii) new scientific findings emerging from the trial's intermediate analyses or from external studies, providing evidence of efficacy for one of the treatments.

Once the trial has started:

- If any experimental treatment proves to be efficacious before the end of the trial (via external data or a major sign of efficacy in the trial), the opinion of the DSMB shall be urgently sought and the design adapted to this new context.
- Groups which are experimental shall be interrupted prematurely if the efficacy or futility criteria are fulfilled before the end of the trial, or if the DSMB considers that safety has not been assured.
- New experimental groups can be added, if new evidence suggest that other treatments should be tested.

4.3 Judgement criteria

4.3.1 Main criterion

The main criterion shall be the proportion of participants with an occurrence of hospitalisation and/or death between Day 0 and Day 14 in each arm.

All hospitalisations shall be taken into account, whatever may be their reason. Hospitalisation criteria, however, shall be standardised and specified in a specific procedure [SDPM].[1]

4.3.2 Secondary criteria

- Hospitalisation proportion and its causes between Day 0 and Day 28 within each group
- Proportion of deaths and their causes between Day 0 and Day 28 within each group
- Intensive care hospitalisation proportion and its causes between Day 0 and Day 28 within each group

[1] Currently, the following criteria are being proposed: Sp02<92% in ambient air or 4% reduction in SpO2 since inclusion; respiratory rate >30 min; heart rate >130/min; systolic blood pressure <90 mmHg or diastolic <60 mmHg; other parameters indicating hospitalisation, in the judgement of the attending physician (for example, unexplained fall, severe digestive problems, reoccurrence or persistent fever >38.5°C etc.)

- Proportion of patients with a reversion of SARS-CoV2 RT-PCR on a nasopharyngeal swab on Day 7 and Day 14, in each group
- Proportion of patients with a loss of autonomy evaluated by ADLs and IADLs on Day 14 and Day 28
- Changes in CBC, inflammatory markers (please see paragraph **Erreur ! Source du renvoi introuvable.**) and immunological markers (please see paragraph **Erreur ! Source du renvoi introuvable.**) from Day 0 to Day 7 and Day 14 in each group
- Number and proportion of adverse events of grades 1, 2, 3 ,4 between Day 0 and Day 14 in each group
- Number and proportion of adverse reactions of grades 1, 2, 3 ,4 between Day 0 and Day 14 in each group
- Plasma trough concentration of experimental drugs in the study on Day 7
- Acceptability of the treatment

4.4 Randomisation

There shall be two randomisation lists:

- One list for people not taking renin-angiotensin system blockers (ACEI or ARB) before inclusion.
- One list for people under treatment with renin-angiotensin system blockers (ACEI or ARB) before inclusion.

The randomisation lists shall be created by a statistician at the Clinical Trail Unit (CTU) before the start of the research project. The number of participants in the treatment groups shall be equalised by using a ratio of 1:1 in each arm.

Randomisation shall be stratified by place of residence (home or nursing home). Other stratification factors could be defined over the course of the study by the SAB, if they are deemed necessary.

A document describing the preparation of the randomisation list shall be kept confidential with the CTU. A validated, web-based system shall be used to implement the random allocation to each group.

5 METHODS

5.1 Eligibility criteria

5.1.1 Inclusion criteria

- Positive test for SARS-CoV-2 on a nasopharyngeal swab
- Appearance of symptoms <72 hours before taking the nasopharyngeal swab
- Age ≥ 65
- Valid person, ambulatory, with full capacity to understand the challenges of the trial
- No hospitalisation criteria according to current guidelines
- Signed, informed consent

5.1.2 Non-inclusion criteria

5.1.2.1 Autonomy

- Incapable of understanding the meaning of participating in the study (*i.e.*: dementia) or to make a decision to participate (*i.e.*: person under protection of justice, tutorship or guardianship).
- Person with a score ≥7 on the Rockwood Clinical Frailty Scale
- Chronic illness at a severe stage, with limited life expectancy.

In the particular case of people resident in a nursing home, opinion on inclusion from the coordinating physician of the facility shall be sought, with particular consideration to the environment and the practices at the nursing home.

5.1.2.2 Cardiovascular precautions

- An ECG shall be systematically carried out before inclusion.
  - In the case of known Long QT Syndrome, or QTc interval ≥ 500ms in the ECG carried out before inclusion, that person shall not be included in the trial.
  - In the case of a QTc interval between 460 and 500ms in the ECG carried out before inclusion, or any significant anomaly in the ECG such as repeated ventricular extra-systoles, or any significant cardiovascular history giving grounds to believe a predisposition to rhythmic or conduction abnormalities, where the taking of study drugs could bring about conduction or rhythm problems, cardiological opinion shall be systematically sought before any decision to include [SDPM]. If the opinion is such of being capable of inclusion, that person shall be

subject to daily 6 lead ECG monitoring during the first seven days of treatment (please see paragraph **Erreur ! Source du renvoi introuvable.**).

- Blood electrolytes are not to be systematically analysed before inclusion. On the other hand:
  - If the person has benefited from blood electrolytes testing before inclusion (for example, because s/he has sought urgent consultation with symptoms leading to a blood electrolytes prescription), s/he shall not be included if serum potassium were >5.5 mmol/L or <3.5 mmol/L.
  - The results of the blood electrolytes testing performed at the time of inclusion (please see paragraph **Erreur ! Source du renvoi introuvable.**) shall be sent on immediately to the researchers. In cases of significant hypokalaemia, an appropriate decision shall be made in consultation with the researcher, cardiologist and attending physician.

5.1.2.3 Drug intake

- Treatment in progress or taken in the last 30 days by one of the following drugs: piperaquine, halofantrine, dasatinib or nilotinib.
- Treatment in progress by: citalopram, escitalopram, hydroxyzine, domperidone, powerful inhibitors of the CYP3A4 isoenzyme of the P450 cytochrome (protease inhibitors of HIV or HCV; azole antifungals; macrolides), powerful inducers of the CYP3A4 isoenzyme of the P450 cytochrome (dexamethasone, phenytoin, fosphenytoin, carbamazepine, oxcarbazepine, eslicarbazepine, rifampicin, rifadin, phenobarbital, primidone, millepertuis).

5.1.2.4 Other medical histories

- Known hypersensitivity to one of the trial drugs or to one of its excipients, as well as to one of the following compounds: chloroquine and other 4-amino quinoleines, amodiaquine, mefloquine, glafenine, floctafenine, antrafenine, ARB.
- Presence of one of the following illnesses reported by the person: hepatic porphyria, known obstructive hepatobiliary disease, liver failure (score ≥ Child-Pugh B), chronic kidney disease stage 4 or 5 (GFR <30 mL/min/1.73 m²) or dialysis, retinopathy, lactose hypersensitivity, anomalies in galactose metabolism, lactase deficiency, malabsorption syndrome, known glucose-6-phosphate-dehydrogenase deficiency, symptomatic gout/hyperuricemia.

5.2 Trial treatments

The trial treatments shall be as follows:

5.2.1 Vitamins

- AZINC Forme et Vitalité®
- 2 tablets *QD* from Day 0 to Day 9
- To be kept below 25°C

5.2.2 Hydroxychloroquine

- Brand name: Plaquenil®
- 200 mg per tablet
- 2 tablets *BID* on the day of inclusion (Day 0), then 2 tablets *QD* from Day 1 to Day 9
- To be kept below 25°C

5.2.3 Imatinib

- Brand name: Imatinib TEVA®
- 400 mg per tablet
- 1 tablet *QD* from Day 0 to Day 9 (Day 0: at any time after inclusion; from Day 1 to Day 9: to be taken at 08:00 am)
- To be kept below 25°C

5.2.4 Favipiravir

- Brand name: Avigan®
- 200 mg per tablet
- 12 tablets *BID* on Day 0, then 6 tablets *BID* from Day 1 to Day 9
- To be stored at below 25°C

5.2.5 Telmisartan

- Brand name: Micardis®

- 20 mg per tablet
- 1 tablet per day from Day 0 to Day 9
- To be kept below 25°C

5.2.6 Concomitant drugs

Drugs whose taking is indicated as a criterion for non-inclusion (please see paragraph **Erreur ! Source du renvoi introuvable.**) shall be contra-indications of prescription over the 10 days following inclusion.

In all the arms, the usage rules for additional drugs that could be taken for symptomatic purposes, including paracetamol, shall be explained. A prescription for such treatments could be drawn up by the Doctor of the mobile study team, in close collaboration with the attending physician. For participants without any possibility of having third parties collect these drugs from the chemist's, the mobile study team, in collaboration with the attending physician, shall make the appropriate provisions.

5.2.7 Experimental drugs circuit

The drugs shall be ordered, received and kept at the CHU de Bordeaux - Pellegrin Hospital pharmacy. They shall be re-labelled on their original commercial packaging with a label in compliance with ICH recommendations on clinical research.

Drugs provided in the context of the trial shall be prescribed by the researcher by means of a prescription and a copy of that prescription shall be kept at the pharmacy [SDPM].

All drug containers, full, empty or opened, shall be returned to the pharmacy by the medical team, then kept until the monitoring visit of the pharmacy by the Clinical Research Assistant (CRA) for the trial. No destruction can be carried out without the written authorisation of the sponsor.

One or more monitoring visit(s) of pharmacies shall be carried out by the CRA for the trial.
During this/these visits, the CRA is to verify:

- the conditions under which the drugs are stored (temperature, humidity).
- the methods of dispensing experimental drugs.
- reconciliation between those experimental drugs received, in stock, dispensed and the units of returned experimental drugs.
- documentation for the trial.

5.3 Progress of the trial

5.3.1 Overview

The trial team is to implement mobile medical teams. These teams are to collaborate closely with the attending physicians, the coordinating physician of the nursing homes, medical call centres, diagnostic centres, and emergency services operating in Bordeaux and its surroundings, SOS Doctors and Centre 15.

Participants are to be recruited and monitored according to the following schedule:

Table 1: Schedule of visits

	**Inclusion Day 0**	**Day 1 (1)**	**Day 3 (1)**	**Day 5 (1)**	**Day 7**	**Day 9 (1)**	**Day 12 (1)**	**Day 14 (1)**	**End (1) (2)**
**Clinical examination**									
**Main study**									
Information									
Physical examination	X				X			X	X
Electrocardiogram **(3)**	X				**(4)**				
Sp02 measurement	X				X				X
Eligibility criteria	X								
Signature of consent	X								
Randomisation	X								
Distribution of the drugs	X								
Clinical appointment		X	X	X		X	X		
Receipt of AR/SAR	X	X	X	X	X	X	X	X	X
**Acceptability sub-study (6)**									
Appointment			X	X		X			

**Swab**									
**Main study**									
Nasopharyngeal swab					X			X	
Blood sample	X				X			X	
Volume of blood sampled (ml)	*10*				*10*			*10*	
**Immunological sub-study**									
Blood sample	X				X			X	
Auto-test (drop of blood)	X	X	X	X	X	X	X		
Volume of blood sampled (ml)	*3*				*3*			*3*	

(1) Telephone call.

(2) On Day 28, for people never hospitalised, 7 days after date of discharge from hospital, if not.

(3) The table indicates the ECGs carried out systematically at home by the mobile team. Other ECGs can be carried out in the interim, upon the referring cardiologists' indication, by means of a self-testing remote device

(4) Only those participants taking drugs which have any potential effect on the QT interval or those presenting a QTc between 460 and 500ms on the first electrocardiogram.

5.3.2 Period before inclusion

In this study, there is no pre-inclusion phase. All that occurs ahead of inclusion concerns diagnosis of COVID-19. The nasopharyngeal test shall be carried out in accordance with current recommendations. No examinations, which could not be justified by the person's clinical condition, shall be carried out.

People presenting with signs or symptoms resembling COVID 19 and not having immediate hospitalisation criteria may benefit from a SARS-CoV-2 nasopharyngeal test, upon request:

- *Outside the home*: in all places where the test shall be held at the time of the trial commencing: A&E departments of hospitals or Bordeaux clinics and specific SARS-CoV-2 screening centres, which have been identified earlier.
- *At home and at nursing homes*: the mobile medical teams shall be available for all requests to attend the home address and carry out a nasopharyngeal sample, at the request of the person concerned and subject to the indication that such a sample complies with those recommendations in effect.

People who would benefit from a nasopharyngeal swab shall be informed of the existence of the trial. They shall be sent a notice informing them of what will occur if the test is positive, as well as, if they so wish, the trial information notice. If they are in an emergency department, following appraisal by the clinician, they shall then return home pending results of the test, or shall remain under observation.

During this period when the person has not yet been included, the usual examinations may be decided upon by the medical team taking care of the person in the context of the emergency services (e.g. blood electrolytes in the case of diarrhoea, ECG in the case of cardiovascular risk factors, etc.). Such examinations shall not form part of examinations linked to the trial, but their results could be used while deciding upon inclusion (please see the non-inclusion criteria, paragraph **Erreur ! Source du renvoi introuvable.**).

5.3.3 Inclusion (Day 0)

If the test is positive, the process leading to inclusion and inclusion itself shall take place: At the outpatient COVID-19 screening unit or in A&E, if the person is still there at the time of the results becoming available; by visit by the mobile medical team to the home or Nursing Home, if the person has returned home or lives in the Nursing Home and agrees to being seen by the team.

The test result shall be explained to the person, who shall be invited to participate in the trial. If the person is interested, the conditions for inclusion shall be checked with him/her (including the review of all treatments in progress, a systematic ECG and recourse to cardiological opinion or the on-call cardiologist for the trial for persons on each occasion that it is appropriate).

If the criteria for inclusion have been met and there is no criterion for non-inclusion, all the questions on the trial shall be answered and they shall be asked whether they agree to participate. If they agree, they are to sign with informed consent and are to be included.

After signing the consent, the inclusion visit shall include:

- Physical examination
- Pulse oxygen saturation (SpO2)
- Blood sample
- Randomisation

- Drug delivery
- Advice

Participants are to receive a document explaining what they must do in the event of symptoms suggesting a worsening of the disease or any side-effect of the drug. During the inclusion visit, particular attention is to be drawn to the living conditions of solitary people, their environment in matters of food and access to drugs other than those delivered in the context of the trial and their ability to gain access to the telephone. In the event of any problem with one of these points, suitable solutions are to be found [SDPM].

People with a QTc between 460ms and 500ms upon inclusion, or any other risk factor requiring cardiological opinion before a decision on inclusion, shall be referred to a 6 lead ECG (Kardia Mobile 6L from AliveCor; CE N°72133041) daily monitoring and shall receive the necessary advice on its functioning during the inclusion visit. This user-friendly device allows transmission of the ECG results and remote reading by the research cardiologist for the trial.

5.3.4 Remote monitoring of the ECG and consideration of the blood electrolytes for inclusion

- People in receipt of an ECG device are to take a reading 3-4 hours after the morning treatment intake on Day 1, then daily 3-4 hours after it up to Day 7. The ECG shall be transmitted remotely. The ECG shall be read directly by the on-call cardiologist. In the event of any anomaly, requiring a stop to the trial's drug, the person shall be informed by telephone and receive a visit from the mobile team.

  The device shall be recovered by the mobile team on Day 7.
- The kalaemia dosed on the inclusion sample shall be made available to the researchers on the same day of inclusion. In the event of severe hypokalaemia, the researcher shall contact the attending physician for corrective measures. If the confirmed figure indicates the trial drug should be interrupted, provisions shall be made by telephone with the person, the attending physician and the mobile team, so that the decision can be enacted immediately.

5.3.5 Remote visits (Days 1, 3, 5, 9 and 12)

- Remote visits shall be carried out by means of the web software allowing video contact, if the participant has an internet connection and the possibility of using it in the appropriate manner; or by telephone if the opposite applies. If the person cannot be connected, the mobile team shall be activated.
- Such visits are to be standardised and refer to:
  - Clinical condition (including the recording of undesired effects)
  - Taking of the drugs (trial drugs and concomitant treatments)
  - Carrying out of self-tests for the immunological sub-study (if applicable)
  - Mental state
  - Advice

5.3.6 Face-to-face visits (Day 7 and Day 14)

- Face-to-face visits shall be carried out in the participant's home by the trial's mobile team.
- Face-to-face visits are to consist of:
  - Interview and clinical examination (including the recording of undesired effects)
  - Taking of drugs
  - SpO2
  - Blood sampling
  - Nasopharyngeal swab for SARS-CoV2 RT-PCR
  - Advice

5.3.7 Out of phase contacts during the trial

When any participant calls the telephone number of the trial's point of contact at unscheduled times:

- If there are suspicions that the illness requires a face-to-face appraisal, the mobile team will ensure that a home visit occurs rapidly. This visit could be carried out by the attending physician, the trial's mobile team, or any other health worker authorised to perform home visits.
- If there are suspicions that the illness requires urgent hospitalisation, the team is to contact the emergency services to organise the transfer to hospital [SDPM].

5.3.8 Visit at the end of the trial (Day 28 or later)

- This is to take place:
  - On Day 28 for patients never hospitalised

    - 7 days after the date of discharge from hospital if not
- The visit at the end of the trial shall be carried out by means of the web software allowing video contact, if the participant has an internet connection available and is capable of using it in the appropriate manner; or by telephone if the opposite applies.
- The visit at the end of the trial shall be devoted to:
    - Clinical condition (including the recording of undesired effects)
    - Taking of drugs
    - Mental state
    - Discussion on the progress of the patients' convalescence
    - Advice on the remaining days of convalescence
    - Information on receipt of the trial results

5.3.9 Changes to scheduled visits

With consideration to the tense context in which this study could occur, the trial's team has identified, during its risk appraisal, the risk that the workload linked with the epidemic and the changes in stocks of protection equipment and tests would cause changes in the schedule.

The schedule of home visits could be adapted, without calling into question the quality of the medical follow-up of people participating in the trial. The possible scenarios are as follows:

- The visits on Day 7 and Day 14 could be replaced by remote visits for people available by telephone and whose changes are clinically reassuring. The corresponding samples would therefore be omitted. If visits need to be limited, Day 7 visits shall be maintained as a priority over Day 14.
- The nasopharyngeal tests of Day 14 could be omitted for people, who had tested negative on Day 7.
- Movement of people to the outpatient diagnostic centre of the CHU de Bordeaux (Pellegrin site) arranged for this purpose for the Day 14 visit could be envisaged under three conditions: (i) confinement of the population has been implemented; (ii) these people are prepared to go out and make the visit; (iii) the prevention instructions (wearing of masks, social distancing, transport using personal vehicles) are observed. In such cases, transport could be organised by the trial's mobile teams.

Potential changes to the schedule shall be strictly framed and submitted to the SAB.

5.3.10 Biological examinations

5.3.10.1 Nasopharyngeal swabbing

Nasopharyngeal swabbing is to be carried out upon pre-inclusion (for diagnostic purpose and not part of the trial's procedures), on Day 7 and Day 14, transported and processed in the laboratory according to a standard procedure [SDPM].

5.3.10.2 Blood tests

Blood samples are to be taken on Day 0, Day 7 and Day 14 transported and processed in the laboratory according to a standard procedure [SDPM].

Blood samples are to be used thus:

- The day of sampling (on Day 0, Day 7 and Day 14): CBC, PCT, CRP, ferritin, creatinine, urea, sodium, potassium, chloride, calcium, magnesium, albumin, bicarbonates/tCO2, LDH, CPK, ASAT, ALAT on Day 0, Day 7 and Day 14.
- Remotely, on frozen plasma and kept in biobanks:
    - On Day 7 for later pharmacological examination (measurement of plasma trough concentrations of the drugs by HPLC/MS-MS)
    - On Day 0, Day 7 and Day 14 for immunological examination, if the patient has agreed to participate in the immunological sub-study [SDPM] (please see paragraph **Erreur ! Source du renvoi introuvable.**)

5.4 Management of adverse events and new findings

5.4.1 Definitions

Adverse event (article R1123-46 of the Public Health Code)

Any toxic signs occurring to a person, who is participating in research involving the human body, whether these signs are linked to the research or not or to that product, which is the subject of such research.

Adverse reaction (article R1123-46 of the Public Health Code)

Adverse reaction occurring to a person who is participating in research involving the human body, whether this reaction is linked to the research or not or to that product, which is the subject of such research.

Adverse reaction of an experimental drug (article R1123-46 of the Public Health Code)

Any toxic and adverse reaction to an experimental drug, whatever may be the dose administered.

Serious adverse event or serious adverse reaction (article R1123-46 of the Public Health Code and ICH guide E2B)

Any adverse event or reaction which:

- causes death,
- puts the life in danger of the person submitting to research,
- requires hospitalisation or prolongs that hospitalisation,
- causes a severe or lasting incapacity or handicap,
- leads to a congenital anomaly or malformation,
- or any event deemed to be medically serious,

and pertaining to the drug, whatever may be the dose.

The expression "puts the life in danger" is reserved for an immediate life threatening event, at the time of the adverse event.

Unexpected adverse reaction (article R1123-46 of the Public Health Code)

- For research pertaining to a drug: any adverse reaction to the product of a nature, severity, frequency or change not conforming with the benchmark safety information specified in the summary of product characteristics.

New finding (article R1123-46 of the Public Health Code)

- Any new data likely to lead to a re-evaluation in respect of the benefits and the risk of the research or of the product, which is the subject of the research, to modifications in the use of this product, in the conduct of the research, or documents relating to the research, or the suspension or the interruption or the change in protocol of the research or of similar research.
- For trials pertaining to the initial administration or use of the health product for people presenting no illness: any serious adverse reaction.

5.4.2 Description of expected adverse events

Expected adverse events for the trial's drugs are those specified in the Summary of Product Characteristics (SPC) for Plaquenil®, Imatinib TEVA®, Avigan® and Micardis®.

The Summary of Product Characteristics (available online on the trial eTMF) provides the complete list and the level of risk for each drug. The risks of adverse reactions for the 4 experimental drugs for short treatments of 10 days are slim. Adverse reactions, whether the most frequent (even if they are benign) or the most serious (even if they are rare) are as follows:

- Hydroxychloroquine: abdominal pain, diarrhoea, nausea, vomiting, lengthening of the QT interval and *torsades de pointes* or ventricular tachycardia, hypersensitivity, medullar aplasia, pruritus, rash, headache and hypoglycaemia.
- Telmisartan: rash, orthostatic hypotension, cardiac decompensation, hypersensitivity and elevation of transaminases.
- Imatinib: cardiac decompensation, elevation of transaminases, neutropenia, thrombocytopenia, anaemia, pancytopenia, headache, dyspnoea, cough, abdominal pain, vomiting, nausea, diarrhoea, cramps and water retention.
- Favipiravir: elevation of transaminases, diarrhoea, nausea, vomiting, headache, hyperuricemia and hypertriglyceridemia.

Any adverse event, neither appearing in the list of expected events, nor in the latest version of the benchmark document, is deemed to be unexpected.

5.4.3 Conduct to be maintained by the researcher in the case of adverse event or new finding

5.4.3.1 Reporting of adverse events (AE)

The researcher is responsible for reporting adverse events occurring between the date of signature of consent and the end of the participant's participation, apart from those detected before the first application of the trial's treatment. If the clinical or biological adverse event is present at the time of signature of consent, only its deterioration needs to be recorded.

In the context of this protocol, all adverse events must be reported.

The researcher is to record adverse events (clinical and biological) in the observations log. Such adverse events are researched upon every visit over the course of the study by the posing of questions and during the participant's clinical examination.

5.4.3.2 Immediate notification of serious adverse events (SAEs) and new findings

The researcher is to evaluate each adverse event with regard to its seriousness.

The researcher must notify the Safety and Monitoring Unit by fax, email or via the eCRF [SDPM], **immediately** from the day of becoming aware of it, of all SAEs and all new findings, with the exception of the following SAEs*:*

- Pre-existing or documented pathology before the signature of consent to the trial and remaining at a degree of severity equal or lesser than that preceding the initial treatment of the trial.
- Hospitalisation for medical or surgical treatment scheduled before the research;

If the researcher becomes aware of a Serious Adverse Event, for which he suspects a causal link with the research, occurring after the end of research for any participant whom he has treated, he is to inform the Safety and Monitoring Unit immediately.

The researcher must document the event to the best of his ability, providing, if possible, the medical diagnosis. The researcher must satisfy himself that information relevant to the follow-up has been communicated to the Safety and Monitoring Unit as soon as possible.

The researcher must send on, in addition to the form for notifying SAEs, copies of the laboratory results or examination or hospitalisation reports, giving details of the Serious Adverse Event, including the relevant negative results **without omitting to anonymise these documents** and include the participant's number and code before sending.

The researcher must monitor the participant having presented with an SAE until its resolution, stabilisation at a level judged by him as medically acceptable or the previous state resuming, even if the participant has stopped the research's procedure. Any addition to the information pertaining the changes in the event, if it has not been mentioned in the first report, shall be sent to the Safety and Monitoring Unit by the researcher.

All Serious Adverse Events, for which the researcher or the Safety and Monitoring Unit deems that a causal relationship could plausibly be foreseen, are to be considered as suspicions of Serious Adverse Reactions.

5.4.3.3 Recapitulating table of the flow of notifications per event type

EVENT TYPE	NOTIFICATION METHODS	NOTIFICATION SCHEDULE TO THE SAFETY AND MONITORING UNIT
**Non-serious** adverse event	AE form in the observations log	No immediate notification
**Serious** adverse event	"AE" form in the observations log and initial SAE declaration form (+ Follow up, if necessary) + written report, if necessary	**Immediate notification**
New finding	Form for sending out an alert (EN-RCL-515) and form "AE" from the observations log, if applicable	**Immediate notification**

Safety and Monitoring Unit for clinical research - CHU de Bordeaux

Tel: +33 5 57 82 16 26

Fax: +33 5 57 82 12 62

Email: vigilance.essais-cliniques@chu-bordeaux.fr

5.4.4 Declaration by the sponsor of Suspected Unexpected Serious Adverse Reactions and new findings.

The Safety and Monitoring Unit is to evaluate if the Serious Adverse Reaction is expected or unexpected, while using the benchmark document as its basis (SPCs of the trial's drugs).

The Safety and Monitoring Unit is to declare to the relevant authorities any Suspected Unexpected Serious Adverse Reaction (SUSAR) occurring in France and outside the national territory and within the following timescales:

- 1). In the case of the SUSAR having caused death or threatened life, immediately from the day of the sponsor becoming aware of it,

– 2). In the case of other SUSAR, at the latest within a timescale of 15 days from the day of the sponsor becoming aware of it.

The Safety and Monitoring Unit is to declare in the form of a follow-up report to the ANSM that relevant additional information regarding the SUSARs:

– In the case of suspicion of SUSAR, having brought about death or threatened life, such information is to be notified within a timescale of 8 days from the time of the declaration mentioned in point 1)
– In other cases of SUSAR and for new findings, relevant additional information is to be sent within a new timescale of 8 days from the timescale mentioned in point 2).

Pertaining to any drug study, the Safety and Monitoring Unit is to record on the EudraVigilance database all SUSARs.

The Safety and Monitoring Unit is to declare immediately any new findings occurring over the course of the research:

– to ANSM,
– to the Institutional Review Board.

The sponsor and the researcher are to take the appropriate urgent measures. The sponsor shall inform the relevant authority and the Institutional Review Board of such.

5.4.5 Annual safety report

On the anniversary date of the research's authorisation, the Safety and Monitoring Unit shall draw up a safety report comprising:

– the list of Serious Adverse Reactions likely to be linked to experimental treatments of the research, including the expected and unexpected Serious Adverse Reactions, occurring in the relevant trial over the period covered by the report,
– a concise and critical analysis of the safety of participants taking part in the research.
– the summary table of all Serious Adverse reactions occurring during the relevant trial since the start of the research.

This report is to be sent to ANSM and the IRB within 60 days after the anniversary date of the research's authorisation.

5.5 Safety of staff

Given the risk of transmission of SARS-CoV2, this study raises questions of safety. The researchers shall give particular attention to the following points:

– Research staff taking part in the study shall be informed of the nature of the research, including the personal risks and the need to follow strict prevention procedures [SDPM].
– Research and care staff taking part in the study shall be trained in standard international procedures for the prevention of SARS-CoV2 transmission and shall be supplied with international standard equipment before coming into contact with the patients. The non availability of adequate equipment shall lead to a revision of the procedures.
– The participants in the trial and their relatives shall receive an explanation of those precautions to be taken to prevent transmission.

6 Statistical analysis

– Inclusion and exclusion from statistical analysis

Participants who died, were lost to follow-up or withdrew consent will be included in the analysis. Participants with at least one of the following conditions could be excluded:

- Having never taken trial treatment or ignoring the allocated arm
- Included without signing a consent form
- Included despite non-compliance with at least one major eligibility criterion
- Having withdrawn consent

The SAB will review cases documentation collected by the trial team and will take an exclusion decision blinded of the randomisation arm and the participant's post inclusion evolution.

Major deviations from eligibility criteria are defined as any deviation on the following criteria:

- Positive SARS-CoV-2 RT-PCR
- Age ≥ 65 years

Randomisation treatment discontinuation will be managed as follows:

- On intention to treat (ITT)

  Any participant will be analysed in the allocated arm, disregarding eligibility deviations or non-compliance with randomisation treatment.

  All data will be used.

- On treatment (OT)

  Any participant will be analysed in the allocated arm, irrespective of eligibility deviations or non-compliance with randomisation treatment.

  All data observed after discontinuation of randomisation treatment will be dropped.

- Per protocol (PP)

  Any participant will be analysed in the allocated arm, irrespective of minor eligibility deviations or minor non-compliance with randomisation treatment.

  All data from any participant with a major eligibility deviation or a major non-conformity with randomisation treatment will be dropped.

- As treated (AT)

  Any participant will be analysed with respect to the actually received treatment, irrespective of the allocated treatment.

Missing values will be managed as follows:

- Analysis on available data (AD): any missing value will be ignored.
- Missing as failure (M=F) procedure: any missing value will be analysed as a failure (main endpoint: disease worsening)
- Worst case procedure (WC):

  First sub-analysis: Any missing data is analysed as a success in the first arm, and as a failure in the second arm.

  Second sub-analysis: Any missing data is analysed as a failure in the first arm, and as a success in the second arm

  The conclusion will be that of each sub-analysis if they are identical.

- Inverse probability weight (ITW) procedure:

  Weighting of values by estimated probability that a value is missing, obtained through modelling from baseline participants' characteristics and follow-up characteristics collected for all participants.

6.1 Principles

The primary endpoint is the by-arm rate of hospital admission or death between D0 and D14 (binary outcome in the primary analysis)

The primary endpoint analysis consists in multiple sub-analyses with multiple arms, multiple stages (sequential analyses) and procedures to manage randomisation treatment discontinuation or missing values. Tests multiplicity due to multiple stages will be managed by alpha spending function methods.

This trial is an adaptive phase III trial. Sequential analyses will be performed for the primary endpoint at D14 (and D28 for the last one) with different objectives:

- Interim analysis n°1: futility at D14
- Interim analysis n°2: futility at D14
- Interim analysis n°3: futility and early efficacy at D14
- Primary analysis: demonstrated efficacy at D14
- Sensibility analysis: maintained efficacy at D28

Each analysis on the primary endpoint will be on intention-to-treat with "missing as failure" (ITT-M=F).

Each experimental arm will be independently compared to the control arm, which leads to several paired tests.

Management of withdrawal or addition of an arm during trial:

- Arm withdrawal

  If an interim analysis demonstrates that it is useless to continue with an experimental arm compared to the control arm, this arm is discontinued and the trial continues with the other arms.

If an interim analysis demonstrates the early efficacy of an experimental arm compared to the control arm, this arm becomes the new control arm and the former control arm is discontinued. Either the SAB and the sponsor decide to definitively stop the trial, or a protocol amendment describes the implications regarding trial conception and statistical analysis, especially the comparison between the new control arm with the other arms.

- Arm addition

  If a new medicine is deemed interesting after the trial started, a new arm is added. A protocol amendment describes the implications trial conception and statistical analysis, especially the comparison between the new arm with the control arm.

The primary analysis will be performed in the strata of participants not taking renin-angiotensin system blockers (ACEI or ARB) before inclusion, if the Telmisartan arm is maintained up to the end of the trial.

6.2 Sample Size

6.2.1 Participants not taking ACEI or ARB before inclusion

The incidence assumptions on primary endpoint are based on extrapolations from aggregated data published in other contexts thus far. The DSMB will check these assumptions and recommend a sample size revision, if needed.

We assumed that the proportion of participants reaching the primary endpoint at D14 in the control arm could be up to 25%. An experimental treatment will be deemed efficient is this proportion is reduced by at least one half. We also considered a power of 80% for all tests.

Three futility (interim) analyses will be performed successively. We defined successive arm discontinuation thresholds for the one-way first type error alpha using STATA package NStageBin for conception and sample size of MAMS trials (65,66).

Two efficacy analyses (interim n°3 and primary) will be performed successively. We will use the O'Brien & Fleming procedure to define the first type error alpha for each analysis, based on the data observed at the first futility analyses (67,68).

Sensitivity analysis at D28 will be exploratory only, without impact on sample size.

Tests of difference between arms will be one-sided. Arm discontinuation thresholds and sample size for each analysis are given here below:

Analysis	**Trigger**<br>Number of participants in the control arm having reached D14	**Objectives**	**Alpha**
Interim n°1	30	Futility	0,65
Interim n°2	60	Futility	0,45
Interim n°3	102	Futility	0,25
		Early efficacy	O'Brien-Fleming
Primary	169	Efficacy	O'Brien-Fleming
Sensitivity	End of trial	Efficacy	0,05

The sample size required is 169 participants by arm (845 overall). Since the "Missing as failure" procedure is used, there is no need to increase sample size to account for missing data.

This size may vary depending on the discontinuation or addition of arms during trial:

- If one arm is discontinued: from -67 participants (discontinuation at interim analysis n°3) to -139 participants (discontinuation at interim analysis n°1)
- If one arm is added: from +169 participants (addition at the time of first participant inclusion) to +338 participants (addition after the last planned inclusion).

6.2.2 Participants taking ACEI or ARB before inclusion

The Telmisartan arm is not assessed in this stratum. The 3 other arms only will be compared. We specify no sample size a priori. The proportion of persons taking ACI or ARB is assumed to be 20% of the target population. Consequently, we expect to recruit 53 participants per arm (212 in total).

Following futility or efficacy analyses, the DSMB may recommend to increase the size to reach a sufficient power for clinically relevant statistical tests.

6.2.3 Overall sample size

Summing the sizes of both strata, and if all the arms go to the end of the trial, we get a final and overall size of 1057 participants. It is likely that one or more arms will be discontinued for futility and the number of participants will not reach this amount.

6.3 Analysis plan

A separate detailed statistical analysis plan will be written and validated by the SAB. Possible future modifications will lead to amendments written and validated before database freezing.

6.3.1 General information on the statistical techniques used

Qualitative outcomes will be described in tables and graphs as follows: frequency, proportion, standard deviation of the proportion, two-sided 95% confidence interval. Comparisons between arms will use a Chi^2 test or an exact Fisher's test (depending on the outcome distribution) if no adjustment is required or logistic or binomial regression otherwise.

Normality of quantitative outcomes will be checked graphically.

Normally distributed quantitative outcomes will be described in tables and graphs as follows: frequency, mean, standard deviation of the mean, two-sided 95% confidence interval. Comparisons between arms will use a Student's t test if no adjustment is required or a linear model otherwise.

Quantitative non-parametric outcomes will be described in tables and graphs as follows: frequency, median, range and interquartile range. Comparisons between arms will use a Wilcoxon or a Kruskal-Wallis test (depending on the number of strata) if no adjustment is required or a stratified analysis otherwise.

Time-to-event outcomes will be described in tables and graphs as follows: number, probability of event and two-sided 95% confidence interval estimated by the Kaplan-Meier method. Comparisons between arms will use a logrank test if no adjustment is required or a proportional risk (Cox) model otherwise.

Model assumptions will be checked before final adjustment.

Descriptive analyses will be systematically presented globally, by country, stratum and arm.

Comparisons between arms will be systematically performed:

- Without adjustment
- With adjustment on randomisation stratification factors
- With adjustment on randomisation stratification factors and on baseline prognostic factors which distribution could be unbalanced between arms despite randomisation.

Moreover, these adjustments will possibly imply the use of appropriate models not cited here. Their relevance will be discussed depending on the factors nature and distribution.

Details on secondary and exploratory analyses on the primary endpoint and analyses on secondary and exploratory endpoints will be provided in the statistical analysis plan.

The identification of determinants of events will be performed using univariate and stepwise multivariate selection models, which details will be defined in the statistical analysis plan.

6.3.2 Accrual and follow-up

Inclusions will be described.

Deviations from eligibility criteria and protocol (withdrawals, loss to follow-up, randomisation treatment discontinuation and replacement treatment, visits et phone interviews undone) will be described.

6.3.3 Baseline characteristics

Characteristics of included participants will be described.

Endpoints and changes in endpoints from D0 will be described at each visit (D7, D14 and D28) or phone interview (D3, D5, D9 and D12).

6.3.4 Primary outcome for participants not taking ACEI or ARB before inclusion

The primary endpoint will be described at each visit and phone interview, as a binary outcome and a time-to-event outcome.

The primary analysis is the set of interim and final analyses on the primary endpoint at D14 (futility and efficacy).

Secondary analyses of the primary endpoint will explore variants of the primary analysis, concerning:

- Management of randomisation treatment discontinuation: on treatment, per protocol, as treated
- Management of missing values: worst case, inverse probability weighting
- Primary endpoint at D7

The sensitivity analysis will repeat the primary analysis at D28 to confirm the results of D14.

6.3.5 Primary outcome for participants taking ACEI or ARB before inclusion

If, following a decision from the DSMB, sample size has been increased to reach a power sufficient for clinically relevant statistical tests, the statistical analysis will repeat the one in the stratum of participants not taking ACEI or ARB before inclusion.

Conversely, if the size has remained the same, the analysis will be descriptive only.

In both situations, if the Telmisartan arm shows no efficacy during futility (arm discontinuation) or efficacy analyses in the stratum with participants not taking ACEI or ARB before inclusion, both strata will be pooled in a single analysis.

6.3.6 Secondary endpoints

The analyses on the secondary endpoints will be performed on intention-to-treat on available data, except for safety analysis.

6.3.7 Safety analysis

The analysis will be performed on participants having taken at least one dose of randomisation treatment on available data, from D0 to D14 and D28.

Adverse events will be described through SOC and PT levels of MedDRA classification (last version at time of database freezing).

The incidence of adverse events will be described in terms of nature, causality, severity, intensity.

The incidence of adverse events related to randomisation treatment discontinuation or dosage modification will be described and compared between arms.

Safety analysis will be performed in compliance with the recommendations of the Consort Statement on harm.

7 Governance

7.1 Sponsor

The co-sponsors are to be:

- CHU de for activities occurring within France
- Inserm-ANRS, for activities occurring within Africa (which shall be described in the ancillary protocols).

The Inserm 1219 team has held numerous international studies sponsored by the two sponsors. This common experience guarantees the fastest possible implementation of the study.

7.2 Trial team

CHU de Bordeaux et Bordeaux University, Bordeaux, France:

- Denis Malvy and Xavier Anglaret will be in charge of scientific coordination
- The department of infectious diseases and tropical medicine of CHU de Bordeaux (Thierry Pistone, Alexandre Duvignaud, Arnaud Desclaux, Duc Nguyen) will provide expertise in infectious diseases.
- IDLIC, EUCLID and LEHA of Inserm 1219 Research Center (Edouard Lhomme, Lucile Hardel, Ambre Gelley, Laura Richert, Rodolphe Thiebaut, Linda Wittkop, Olivier Marcy, Joanna Orne Gliemann, Sophie Kacher, Delphine Gabillard, Corine Chazalon, Valérie Journot, Rémi Sitta, Eric Balestre, Catherine Helmer) will be in charge of overall coordination, data management, statistical analysis and methodological expertise under the supervision of the sponsor.

- The laboratory of virology (Marie-Edith Lafon, Isabelle Garrigue et Pascale Trimoulet) will supervise virological analyses.
- The department of pharmacology (Mathieu Molimard; Stéphane Bouchet) will be in charge of experimental drug pharmacokinetics.
- The department of general medicine (Jean-Philippe Joseph, Racha Onaisi) will supervise the network of generale practitioners.
- The department of clinical hematology of Institut Bergonié (François Xavier Mahon) will provide expertise regarding imatinib.
- The department of Emergency Medicine of CHU de Bordeaux (Michel Galinski, Cédric Gil-Jardiné) will supervise the network of emerency medicine departments involved in the trial.
- The department of Cardiology/Hypertension of CHU de Bordeaux (Antoine Cremer, Philippe Gosse) will provide expertise regarding renin-angiotensine-aldosterone system.
- The department of Cardiology/Rythmology of CHU de Bordeaux (Frédéric Sacher) : will provide epxertise regarding rhythm abnormalities risk management.
- The department of acute and neurovascular geriatric medicine of CHU de Bordeaux (Claire Roubaud) will provide expertise regarding geriatric aspects.
- The VILL-HOP COVID platform and Ange Gardien platform (Charles Cazanave, Mathilde Puges, Jean-Luc Pellegrin, Thierry Schaeverbeke) will give support regarding identification and follow-up of eligible patients.
- The pharmacy of CHU de Bordeaux (Sarah Djabarouti, Bellabes Ghezzoul, Céline Plessis) will be in charge of experimental treatments.
- The department of biological ressources of CHU de Bordeaux (Isabelle Pellegrin) will be in charge of biobanking and of performing a part of immunological analyses.
- The direction of clinical research of CHU de Bordeaux (Anne Gimbert, Sébastien Marchi, Pierre Poulizac, Patrick Cassai, Sophie Régueme, Caroline Roussillon, Marine Rousset, Francesco Salvo, Valérie Marty, Sandrine Vautrat) will be in charge of administrative and safety aspects.

AP-HP, Paris Diderot University, Paris Est University, Paris, France :

- The department of infectious diseases and tropical medicine of CHU Bichat-Claude Bernard (Nathan Peiffer Smadja) will be co-investigator of the trial and will ensure the liaison with the DisCoVeRy trial.
- Inserm UMR 1137 unit (France Mentré, Jérémie Guedj) will be in charge of pharmacometric aspects.
- Inserm U955 unit (Christine Lacabaratz, Hakin Hocini) & Vaccine Research Institute (Yves Levy) will supervise immunological aspects.

8 Monitoring

8.1 Scientific Advisory Board

- The Scientific Advisory Board (SAB) is to meet by teleconference every other week.
- It is to be composed of:
  - The coordinating investigator and the research coordinator, the senior methodologist, the senior statistician, the project manager and the monitoring advisor.
  - 3 experts in the areas of infectious diseases, clinical trial and immunological methods and the pharmacological monitoring of clinical trials.
  - One representative for the sponsor and for each partner institution.
- This guarantees that the trial is held in an appropriate manner at the scientific, clinical and ethical levels. That includes:
  - Checking that the team charged with the trial is observing protocol and ensuring the safety of participants
  - Guaranteeing the relevance of the trial’s questions and the validity of its methods.
  - Making decisions regarding those necessary and relevant changes to the protocol.
  - Deciding whether to open or close trial sites.
  - Checking observance of the rules regarding access to data, reports and trial publications.
  - Ensuring liaison with the DSMB and the regulatory authorities.

8.2 Data & Safety Monitoring Board

- The Data & Safety Monitoring Board (DSMB) is to meet by teleconference every other week. In the interim, it shall be informed daily of the number of hospitalised and deceased participants.
- It shall be composed of three external, independent experts in the disciplines of infectious diseases, pharmacovigilance and suitable clinical trial methods, nominated by the sponsor.
- It is to oversee the general running of the trial, in order to protect the safety and interests of the participants. It shall provide advice on the conduct of the trial, pertaining to inclusion rates, quality of follow-up, adverse reactions, interim analyses of the main judgement criterion and recruitment of the required number of participants. It shall assist in making difficult decisions, which require an independent evaluation, while the trial is in progress.
- It shall have access to safety data, as well as any information justifying any change that affects the roll-out of the trial.

9 Sub-studies

Two sub-studies are in preparation. This chapter describes their objectives and methods. They shall be offered to a sub-group of participants. They shall be the subject of a specific consent. They shall only start once the main study is under way and functioning in a manner judged satisfactory by the SAB. The information and consent forms shall be submitted to the IRB for approval before the start of these sub-studies. Given the urgency of implementing the main study, this submission shall be postponed.

9.1 Immunological sub-study

The immunological sub-study has three explanatory objectives:

- evaluating the impact of study drugs on immunological parameters.
- identifying the immune biomarkers associated with clinical worsening.
- identifying the immune biomarkers associated with a change of the effect of those interventions evaluated.

In relation to the main study, it involves two things: (i) sampling of additional blood tubes on Day 0, Day 7 and Day 14 (additional 3ml); (ii) daily sampling of blood drops on a cotton swab (8 in total during the follow-up). This is only a gesture, yet whose enactment could prove difficult for certain people, or undesired by others in this particular context. It shall therefore be carried out by a sample of volunteers and subject to specific consent.

9.1.1 Justification

The initial reports of patients heavily infected by CoV-2 of SARS have shown an increase in pro-inflammatory cytokines associated with severe pulmonary lesions and a transfer to Intensive Care Units (69). Patients infected with SARS CoV and MERS CoV have developed CD4+ and CD8+ T cells as well as B cells specific responses (70). As a result, even though the mechanisms and consequences of the hyperinflammation may be unknown, it is possible to posit the theory that the premature changes in some of these markers could be predictive of future clinical evolution. Furthermore, the reduction in hyperinflammation could be one of the mechanisms for which intervening could have a clinical impact. Finally, the presence of hyperinflammation could influence the effect of intervening, as is the case with IL-1 effect blockade in septicaemia (71).

9.1.2 Methods

As patients are at home, we shall be using a new technology allowing measurement of the whole blood's genetic expression via a single drop of blood on a cotton swab taken by the patient himself from a finger. We have already demonstrated the validity of this approach in terms of RNA quality (RIN 6.9) and in accordance with the measures emerging from sampling by Tempus tube.

The following samples shall be taken:

- Day 0, Day 7, Day 14: 1 ETDA tube, 1 Tempus tube (total blood volume 3ml),
- Day 0, Day 1, Day 3, Day 5, Day 7, Day 9, Day 12, Day 14: 1 cotton swab

Day	0	1	3	5	7	9	12	14
Fresh blood (ETDA)	X				X			X
Serum (dry tube)*	X				X			X
Genes (Tempus tube)	X				X			X

Genes (cotton swab)	X	X	X	X	X	X	X	X

* 1 aliquot shall be reserved on the dry tube of the main study on Day 0, Day 7 and Day 14 for analysis of the functional profile.

9.1.3 Laboratory

Cellular phenotypic profiles: immunogenicity tests shall be carried out on the whole blood.

- Cells CD4+ and CD8+T are to be analysed for CD45RA expression and C-C chemokine receptor type 7 (CCR7), to identify the sub-populations of naive, memory and effector cells, and for the co-expression of activation markers, HLA-DR and CD38.
- The sub-populations of B cells are to be analysed with markers CD19, CD21, CD27, CD38, immunoglobulin (Ig) and IgM. Cells secreting antibodies are to be identified as CD19+ cells, expressing CD38 and CD27.
- The sub-populations of NK cells are to be analysed by means of CD16 and CD56 markers.
- HLA-DR, CD33, CD45RA, CD123, CD141 and CD1c are to be used in identifying DC sub-populations.

Functional profiles, quantification of serum analytes: Th1/Th2/activation/inflammation/apoptose markers are to be measured in serum by a Luminex assay, allowing the detection of more than 40 analytes with a commercial kit, according to the manufacturer's instructions. The Median Fluorescence Intensity for each sample is to be measured by means of the Bio-Plex 200 system (Bio-Rad, Marnes-la-Coquette, France). The Bio-Plex Manager version 6.0 software, which integrates a weighted adjustment of the logistical curves by five parameters, is to be used to calculate the concentrations of samples.

Genetic expression profiles (whole blood sampled by Tempus tube and cotton swab): Genetic expression profiles are to be analysed by RNA sequencing. RNA extraction is to occur according to the Illumina protocol before being sequenced on a HiSeq 2500 V4 system from Illumina. FastQ forms allow the identification of genes differentially expressed by use of normalised reading counts as input for Gene-Specific Analysis (GSA) carried out on the Partek® Flow® and R software.

9.1.4 Scale of the study

The number of people participating in this study is to depend on the take-up rate.

In total, 30 patients/arms with all measures (maybe 150 in total). This scale of sampling allows description of the phenotypic and functional profile of each arm upon inclusion and evolution over time with enough precision (72,73).

In the event of good take-up, the study of the genetic expression profile on cotton swabs could be proposed for all patients included in the main study. 20 to 40 events per treatment group are expected, which would make 100 to 200 events in total at the end of the trial, if all the participants in the main trial agree to participate in this sub-study; possibly 10 to 20 clinical progression predictors are possible with the conservative rule of 10 events per predictor (74).

9.1.5 Statistical analysis

For each treatment arm, cellular phenotypic and functional profiles will be described (absolute count and proportion), at inclusion and over time. The evolution with time will be compared to baseline values using parametric and non parametric tests, taking into account the multiplicity of tests (False Discovery rate).

Inter-measure agreement between the samples made on Tempus tubes and on cotton-sticks on the 30 patients per group will be studied thanks to intra-class correlation and Bland & Altman graphical method.

The evolution of gene abundance over time will be studied separately on the different type of samples. Supplemental sampling points made available by auto-sampling with cotton-sticks will be analyzed according to the first results obtained at D0, D7 and D14. Statistical analysis will be performed i) gene by gene with a functional enrichment analysis of differentially expressed genes (Ingenuity Pathway) and ii) by gene groups (75).

In the sample of patients (30 per group) with all measures available, an integrative analysis will be performed to study the association between gene abundance and other immunological and virological markers.

The association between biomarkers and clinical evolution will be adjusted on genic expression at inclusion and over time as well as on clinical and biological predictors.

9.2 Feasibility and take-up sub-study

9.2.1 Justification

The urgent implementation of the screening, home-treatment and follow-up model foreseen by the trial poses enormous challenges of logistical and human feasibility and gives rise to questions regarding the acceptability of activities undertaken, as much from the point of view of patients as general practitioners, other carers and mobilised research teams. The more often the constraints and opportunities are documented at each stage of the model being implemented, the more corrective solutions can be adopted and lessons learned for the transferability into other contexts. The more the intended model is well-accepted by the different stakeholders, the more it will be likely to be implemented as intended and thus be more effective. The idea of this sub-study is to use the principles of research on the implementation (document the main elements of acceptability and feasibility) to describe rapidly the roll-out of the clinical trial and inform its implementation (76).

9.2.2 Methods

Mixed methods are to be used to provide measures of logistical, human and technical resources; to document patients' and clinicians' experiences, interactions between patients and clinicians, interactions between patients and mobile teams, obstacles/facilitators to treatment and care, the perception of the quality of care; the general context of implementing this model shall also be documented to describe its influence on the effectiveness of implementation and transferability of the model in different contexts. More precisely:

- A short questionnaire on acceptability shall be offered to all participants on Day 3, Day 5 and Day 9;
- A brief individual targeted interview shall be held with 5 to 8 participants on Day 3
- Group discussions are to be organised with the implementation teams in the field, in order to document the daily challenges and opportunities and react quickly to logistical changes.

The quantitative feasibility and acceptability data is especially to be based on:

- Data drawn from the acceptability questionnaire at the participant level
- Process indicators:
    - number of face-to-face visits carried out in relation to the number foreseen (by indicating constraints at the patient or care team level)
    - duration of face-to-face visits
    - duration of patient follow-up
    - changes in the staff involved (number, profile and training)
    - equipment used
    - costs

Qualitative data on feasibility and acceptability shall be analysed according to a pragmatic approach to the method of "thematic analysis", guided by the predefined themes on individual and group interviews. Results are to be summarised according to the main themes and interpreted, in order to formulate recommendations on the implementation of phase III of the trial.

Quantitative data from the questionnaire and qualitative from the interviews shall be integrated into the management, backup and confidentiality system for all trial data.

9.3 Data management

All activities linked with the management and security of data are to be described in the trial's data management plan.

9.3.1 Data management software

The electronic tool for online randomisation has been developed by the Inserm team U1219 on the ASP.NET/SQLServer. This tool and its database are hosted on servers at the Inserm Centre, U1219/ISPED, which are administered by the Centre's IT Department.

The software in use for data management is REDCap. It is linked to a dedicated MySQL database.

IT Systems Management (DSI) at CHU de Bordeaux assures hosting and maintenance of the database in accordance with its procedures. The servers (Internet and database) are hosted within the DSI of the CHU de Bordeaux (Talence, Gironde, France)

9.3.2 Source data

Apart from the biological examination results, patient data shall be entered directly into the e-CRF and shall as a result have no source data.

9.3.3 Data security

Access to the private site for randomisation is secured, controlled and traced; authentication is reserved for team members and the administration of the user accounts assured by the project's IT team. Access to the complete randomisation list is exclusively reserved for the project's statisticians.

All information on participants shall be strictly confidential. Information, such as the participant's name or any other data able to lead to identification of the participant, shall not be recorded in the trial's database.

All paper documents and digital forms necessary for data management are reserved for authorised trial staff.

The trials' database shall only be accessible by means of personal user name and password. Management of access rights to the software is the responsibility of the REDCap administrator at the CHU de Bordeaux. A list of authorised users of the database shall be updated during the study.

9.3.4 Entry and control of data

Entry of data into the eCRF shall be the responsibility of the Centre's researcher. All persons carrying out entries to the eCRF or carrying out requests for online randomisation shall be trained beforehand and delegated by the coordinating investigator to do so. A guide to using the various tools shall be drawn up. Data shall be recorded by means of tablets (mobile teams) and computers (outpatient COVID-19 testing unit). Controls shall be programmed in, so as to check the coherence and completeness of data entered into the eCRF. Prescribed treatments and clinical events are encoded in order to be able to carry out the control and analysis of data.

9.3.5 Control of AE/SAE databases

The crossover of databases shall be supervised by the Safety and Monitoring Unit (SMU) of the CHU de Bordeaux, according to the procedure in effect within the EUCLID team. Comparisons shall be carried out regularly, according to a schedule defined by the DSMB. The Data Manager shall be responsible for sending the table of adverse events to the Safety and Vigilance Monitoring Unit.

9.3.6 Setting the database

The database shall be set for the final analysis. Setting of data is to be carried out in compliance with the procedure in effect.

9.3.7 Archiving of the database

Archiving of the database shall be the responsibility of the study's sponsor. Data from the study shall remain stored on the DSI server at the CHU de Bordeaux, in compliance with the regulations in effect for the study. A physical copy shall be retained by the sponsor in compliance with regulations in effect.

9.3.8 Quality management

The data management plan for the trial and the procedure manual are to describe in detail:

- the system used to digitise the data
- those methods permitting guarantees of restricted access to the database, backup of data and confidentiality
- procedures relating to the titling of data, tracing of data, incident management and best practices in matters of IT security.

9.3.9 Confidentiality and security of participants' data

All information on participants shall be strictly confidential. Information, such as the participant's name or any other data able to lead to identification of the participant, shall not be recorded in the trial's database.

All paper documents and digital forms necessary for data management are reserved for authorised trial staff, as much at the international level as the local. The trials' database shall only be accessible by means of personal user name and password. A list of authorised users of the database shall be held by the CTU and updated during the study.

9.4 Monitoring

9.4.1 Clinical Trial Unit (CTU)

The monitoring team shall comprise of an international CTU and a national CTU by participating country.

The International CTU is to assure international monitoring, including remote data monitoring, on-site visits and the centralised multi-country monitoring, according to a monitoring plan established earlier. It shall be constituted of team members from MEREVA and EUCLID.

The National CTU is to assure monitoring of the country, including remote data monitoring, on-site visits and the centralised country monitoring, according to a monitoring plan established earlier.

The monitoring team is to supervise the roll-out of the trial to verify that:

- The rights, safety and well-being of the trial's participants are protected
- The recorded and analysed data is precise, complete and coherent
- The trial is held in compliance with the applicable protocol, Normalised Standard Operating Procedures (NSOP), Good Clinical Practices (GCP) and regulatory requirements

9.5 Differences and deviations to the protocol

Non-compliance with the protocol, procedures, good clinical practices or laws and regulations in effect by a researcher, a member of the trial's staff, a participant or any other person during conduct of the trial must lead quickly to the implementation of appropriate measures by the sponsor. The sponsor does not authorise any exemptions to the protocol.

The sponsor undertakes to respect the protocol pertaining to the conditions under which the research must be carried out and the communications process and publication of the trial's results.

In the event of deviating from the protocol, the researcher must justify it, document it and inform the sponsor of such as soon as possible.

Differences and deviations in relation to the applicable benchmarks are classed in three categories: (i) *Minor*: Conditions, practices or procedures, which are unlikely to affect the rights, safety or well-being of the subjects or the quality or the integrity of the data; (ii) *major*: Conditions, practices or processes, which are likely to affect the rights, safety or well-being of the subjects or the quality or the integrity of the data; (iii) *Critical*: Conditions, practices or procedures, which affect the rights, safety or well-being of the subjects or the quality and the integrity of the data.

Critical differences are considered as completely unacceptable. If a serious or deliberately repeated deviation by any researcher or other person participating in the research is detected during monitoring, quality control or audit of the research, the sponsor may end the participation of that person in the trial.

At each meeting of the DSMB and the SAB, the international CTU is to produce an exhaustive list of abandonments, withdrawals of consent, interruptions in the trial's strategy and all deviations.

9.5.1 Essential documents

Essential documents for the trial shall be kept in a main trial file (e-TMF), which shall contain all essential technical documents for the trial (protocol, CRF, SOP, data management plan, monitoring plan and statistical analysis plan) and regulatory (ASNM authorisation, IRB approvals, task delegation forms, CVs, participation agreements) according to the ICH list. The e-TMF shall be made available online in a digital version with permanent access to authorised team members. The main researchers at the sites shall be charged with making it accessible to all members of their team, who would require such documents to carry out the study.

Each participating site shall hold the appropriate medical and research files for this trial, in compliance with the regulatory and institutional requirements in matters of protecting confidentiality.

9.5.2 Audits, file inspections

Each site shall permit authorised representatives of the sponsor and regulatory bodies to examine (and, when the applicable legislation so permits, to copy) the clinical files for the purposes of quality assurance, audits and security appraisal, for the advancement and the validity of the study's data. The researcher shall inform the sponsor of any audits foreseen by the regulatory authorities or ethical committees and shall send on copies of received reports.

10 ETHICAL ASPECTS

1.1.1 ETHICAL AND REGULATORY CONSIDERATIONS

The sponsor and the investigators are responsible of the appropriate conduct of the research in accordance with the n°2012-300 du 5 mars 2012 law regarding research perform on human beings as well as the Good Clinical Practices (I.C.H. version 4, November 2016) and the declaration of Helsinki (available on http://www.wma.net).

The research must be conducted in compliance with the present protocol. Appart emergency situations requiring specific therapeutic measures, the investigators commit to comply with the protocol in particular with respect to consent collection and safety monitoring.

This research has been approved by the Comité de Protection des Personnes (IRB) Ile de France I and the ANSM (French Drug Safety Agency).

The CHU de Bordeaux, acting as the sponsor of this research, suscribed a civil liability policy with HDI Global SE, in accordance with the Public Health Code.

The data recorded for this research are subject to a digital processing by EUCLID, under the conditions of the law n°78-17 of January 1978 modified by the law n°2004-801 of August 2004 and in accordance with the General Regulation on the Protection of Data (EU 2016/679).

This research is complying with the reference framework MR-001 in application of the article 54 indentation 5 of the law n°78-17. This change has been approved by the decision of January 5th 2006, updated on July 21st 2016. The structure in charge of data processing has signed an engagement of conformity with this reference framework. This research is registered in the european database EudraCT under n° 2020-001435-27.

This research is registered on http://clinicaltrials.gov/

After the end of the research, the conservation of biocollection will be declared to the Ministry in charge of Research and to the Agence Régionale de Santé (and submitted to the IRB in case the aim of the research has changed).

10.1 Protocol modifications

Every change in this protocol which could have a substantial impact on the protection of the participants, on study objectives or on the methods to be used shall be subject to a written amendment.

The amendment shall describe point by point the proposed changes and their justification. A revised version of the protocol shall highlight those changes. The amendment and a revised version of the protocol shall be approved by the sponsor and by relevant ethical and regulatory authorities of each participating country before they can be fully adopted.

10.2 Confidentiality

The data recorded for the purpose of this study will be subject to a digital processing at Inserm 1219 research center. The sponsor shall declare the database to the Commission Nationale Informatique et Liberté (CNIL), in accordance with the law n° 78-17 of January 6th 1978 modified by the law n° 2018-493 of June 20th 2018.

All the data shall be pseudonymised and each participant will be given a unique identification code. This code will be the only identifier of the participant at every stage of the trial as well as in the database. There will be no link between the participant name and its identifier in the database.

Only people authorized by the sponsor and involved in the trial management or health authorities could gain access to participants' medical files to verify the accuracy of recorded data.

Those identifying data that are necessary for the follow-up of the participants (name, phone number, address, email) will be collected on a paper document by the study mobile medical teams then entered in a follow-up registry secured by a password stored on a server at the CHU de Bordeaux DSI. The access to this space will be restricted to investigators. All the paper documents as well as the digital registry will be deleted at the end of the research.

10.3 Insurance and care of participants

The sponsor and the study shall take out civil liability policy covering those risks encountered by the participants of the trial, in accordance with French law and international regulations. A copy of the insurance certificate shall be available via the e-TMF and accessible to any regulatory authority upon demand.

In Africa, the study's procedures and essential care shall be free of charge for the participants throughout the period of monitoring the study. This includes transport there-and-back to the study centre for each visit to the clinic (routine and unplanned); medicines, tests, visits and hospitalisation, every time that they are requested or approved by the study's medical team.

11 END OF THE STUDY

The official date of the end of the study is the date of the last visit of the last participant.

The sponsor or his representative shall notify the end of the trial to the ethical and regulatory authorities for each country participating within a period of 90 days.

The sponsor, by following the advice of the SAB, DSMB or the ethical and/or regulatory authorities, may decide to finish prematurely. The sponsor and the researchers, in close collaboration with the health authorities of the country, shall take the appropriate measures to guarantee that patients have access to the best care and treatments available, according to those conditions pertaining in each country.

11.1 Publication of results

The international CTU shall analyse the data and present a final, written report to the researchers and the SAB. The researchers shall then draw up the manuscript or the summaries. The final version must be approved by all authors before submission.

Within a period of one year following the end of the research or its interruption, the final report shall be drafted and signed by the sponsor and the researchers. This report shall be made available to the relevant authorites. The sponsor shall send the results of the research to the ANSM and the IRB in the form of a summary of the final report, within a period of one year after the end of the research.

All written or oral communications of the research's results must receive the prior agreement of the coordinating investigator and, if necessary, any committee formed for the research.

Publication of the main results is to mention the sponsor's name, all researchers having included or followed up participants to the research, methodologists, bio-statisticians and data managers having participated in the research and members of the committee(s) constituted for the research. Consideration shall be given to the international rules of writing and publication (The Uniform Requirements for Manuscripts of the ICMJE, April 2010).

Results from the study shall be published independently of the result, positive or negative, of the study.

In accordance with law no. 2002-303 of 4 March 2002, the participants shall be informed, upon their request, of the research's overall results.

11.2 Impact

The expected impact of the COVERAGE trial is as follows

- A contribution to public health response to the current epidemic and the preparation for future epidemics in France and worldwide, including low and middle income countries, by providing high-level proof that outpatient treatment of people affected by SARS-CoV-2 infection and presenting heightened risk factors can reduce hospitalisations and death, that one of these treatments can reduce the time necessary to obtain a negative virological test
- A contribution towards a better understanding of infections due to highly pathogenic coronaviruses by providing original data on the risk of progression towards worsening and its associated factors, into a little-known disease and whose outpatient care has not been codified
- And finally, the availability of therapies to avoid and manage COVID-19 complications among the elderly and patients at risk. French hospitals are overwhelmed by new COVID-19 patients and it is envisaged that beds in intensive care units will very soon be in short supply. Finding an efficacious treatment, which could be administered at home to avoid hospitalisation and transfer to intensive care, is of crucial importance.

11.3 Archiving of documents

All documents essential to the trial included in the TMF shall be kept in a safe place by the trial's researchers and the CTUs, for 15 years after the end of the trial:

- The protocol and the potential changes to the protocol
- The Observation Booklets (copies)
- The source files of those participants who gave their consent
- All other documents and correspondence relating to the research

All such documents are the responsibility of the researcher for the duration of regulatory archiving.

No removal or destruction may be done without the sponsor's agreement. At the end of the regulatory archiving period, the sponsor shall be consulted about destruction. All data, documents and reports liable to be the subject of an audit or an inspection.

12 DATA TRANSFER

Data management is assured by the CTUs. Conditions for the transfer of all or part of the research database are decided upon by the research's sponsor and are the subject on a written contract.

13 BIBLIOGRAPHY

1. Ford N, Vitoria M, Rangaraj A, Norris SL, Calmy A, Doherty M. Systematic review of the efficacy and safety of antiretroviral drugs against SARS, MERS or COVID-19: initial assessment. Journal of the International AIDS Society. 2020;23(4):e25489.

2. Belhadi D, Peiffer-Smadja N, Lescure F-X, Yazdanpanah Y, Mentré F, Laouénan C. A brief review of antiviral drugs evaluated in registered clinical trials for COVID-19. medRxiv. 2020 Mar 27;2020.03.18.20038190.

3. Onder G, Rezza G, Brusaferro S. Case-Fatality Rate and Characteristics of Patients Dying in Relation to COVID-19 in Italy. JAMA. 2020 May 12;323(18):1775–6.

4. Guan W-J, Ni Z-Y, Hu Y, Liang W-H, Ou C-Q, He J-X, et al. Clinical Characteristics of Coronavirus Disease 2019 in China. N Engl J Med. 2020 30;382(18):1708–20.

5. Wu Z, McGoogan JM. Characteristics of and Important Lessons From the Coronavirus Disease 2019 (COVID-19) Outbreak in China: Summary of a Report of 72 314 Cases From the Chinese Center for Disease Control and Prevention. JAMA. 2020 Apr 7;323(13):1239–42.

6. Mo P, Xing Y, Xiao Y, Deng L, Zhao Q, Wang H, et al. Clinical characteristics of refractory COVID-19 pneumonia in Wuhan, China. Clin Infect Dis [Internet]. 2020 Mar 16 [cited 2020 Jun 20]; Available from: https://www.ncbi.nlm.nih.gov/pmc/articles/PMC7184444/

7. Rosenbaum L. Facing Covid-19 in Italy — Ethics, Logistics, and Therapeutics on the Epidemic’s Front Line. New England Journal of Medicine. 2020 May 14;382(20):1873–5.

8. Società Italiana di Anestesia Analgesia Rianimazione e Terapia Intensiva. CLINICAL ETHICS RECOMMENDATIONS FOR THE ALLOCATION OF INTENSIVE CARE TREATMENTS, IN EXCEPTIONAL, RESOURCE-LIMITED CIRCUMSTANCES [Internet]. Società Italiana di Anestesia Analgesia Rianimazione e Terapia Intensiva; 2020 Mar [cited 2020 Jun 20]. Available from: http://www.siaarti.it/SiteAssets/News/COVID19%20-%20documenti%20SIAARTI/SIAARTI%20-%20Covid-19%20-%20Clinical%20Ethics%20Reccomendations.pdf

9. Chan JF-W, Yao Y, Yeung M-L, Deng W, Bao L, Jia L, et al. Treatment With Lopinavir/Ritonavir or Interferon-β1b Improves Outcome of MERS-CoV Infection in a Nonhuman Primate Model of Common Marmoset. J Infect Dis. 2015 Dec 15;212(12):1904–13.

10. Nukoolkarn V, Lee VS, Malaisree M, Aruksakulwong O, Hannongbua S. Molecular dynamic simulations analysis of ritonavir and lopinavir as SARS-CoV 3CL(pro) inhibitors. J Theor Biol. 2008 Oct 21;254(4):861–7.

11. Liu X, Wang X-J. Potential inhibitors against 2019-nCoV coronavirus M protease from clinically approved medicines. J Genet Genomics. 2020 Feb 20;47(2):119–21.

12. Chu CM, Cheng VCC, Hung IFN, Wong MML, Chan KH, Chan KS, et al. Role of lopinavir/ritonavir in the treatment of SARS: initial virological and clinical findings. Thorax. 2004 Mar;59(3):252–6.

13. Lim J, Jeon S, Shin HY, Kim MJ, Seong YM, Lee WJ, et al. Case of the Index Patient Who Caused Tertiary Transmission of COVID-19 Infection in Korea: the Application of Lopinavir/Ritonavir for the Treatment of COVID-19 Infected Pneumonia Monitored by Quantitative RT-PCR. J Korean Med Sci. 2020 Feb 17;35(6):e79.

14. Wang Z, Chen X, Lu Y, Chen F, Zhang W. Clinical characteristics and therapeutic procedure for four cases with 2019 novel coronavirus pneumonia receiving combined Chinese and Western medicine treatment. Biosci Trends. 2020 Mar 16;14(1):64–8.

15. Young BE, Ong SWX, Kalimuddin S, Low JG, Tan SY, Loh J, et al. Epidemiologic Features and Clinical Course of Patients Infected With SARS-CoV-2 in Singapore. JAMA [Internet]. 2020 Mar 3 [cited 2020 Mar 5]; Available from: http://jamanetwork.com/journals/jama/fullarticle/2762688

16. World Health Organization. COVID-19_Treatment_Trial_Design_Master_Protocol_synopsis_Final_18022020.pdf [Internet]. World Health Organization; 2020 Feb [cited 2020 Jun 20]. Available from: https://www.who.int/blueprint/priority-diseases/key-action/COVID-19_Treatment_Trial_Design_Master_Protocol_synopsis_Final_18022020.pdf

17. Chinese Clinical Guidance for COVID-19 Pneumonia Diagnosis and Treatment (7th edition) [Internet]. [cited 2020 Jun 20]. Available from: http://kjfy.meetingchina.org/msite/news/show/cn/3337.html

18. HCSP. Covid-19 : prise en charge des cas confirmés [Internet]. Rapport de l'HCSP. Paris: Haut Conseil de la Santé Publique; 2020 Mar [cited 2020 Jun 20]. Available from: https://www.hcsp.fr/explore.cgi/avisrapportsdomaine?clefr=771

19. Cao B, Wang Y, Wen D, Liu W, Wang J, Fan G, et al. A Trial of Lopinavir–Ritonavir in Adults Hospitalized with Severe Covid-19. New England Journal of Medicine. 2020 May 7;382(19):1787–99.

20. Lescure F-X, Bouadma L, Nguyen D, Parisey M, Wicky P-H, Behillil S, et al. Clinical and virological data of the first cases of COVID-19 in Europe: a case series. The Lancet Infectious Diseases. 2020 Jun 1;20(6):697–706.

21. Beck BR, Shin B, Choi Y, Park S, Kang K. Predicting commercially available antiviral drugs that may act on the novel coronavirus (2019-nCoV), Wuhan, China through a drug-target interaction deep learning model. bioRxiv. 2020 Feb 2;2020.01.31.929547.

22. Lin S, Shen R, He J. Molecular Modeling Evaluation of the Binding Effect of Ritonavir, Lopinavir and Darunavir to Severe Acute Respiratory Syndrome Coronavirus 2 Proteases. bioRxiv. 2020;

23. Hu Z, Song C, Xu C, Jin G, Chen Y, Xu X, et al. Clinical characteristics of 24 asymptomatic infections with COVID-19 screened among close contacts in Nanjing, China. Sci China Life Sci. 2020 Mar 4;

24. The First Affiliated Hospital ZUSoM. Handbook of COVID-19 Prevention and Treatment [Internet]. The First Affiliated Hospital ZUSoM; [cited 2020 Jun 20]. Available from: https://gmcc.alibabadoctor.com/prevention-manual/reader?pdf=Handbook%20of%20COVID-19%20Prevention%20and%20Treatment%20(Standard).pdf&opt=download&version=standard&language=en&content_id=

25. Lack of evidence to support use of darunavir-based treatments for SARS-CoV-2 | Johnson & Johnson [Internet]. Content Lab U.S. [cited 2020 Jun 20]. Available from: https://www.jnj.com/lack-of-evidence-to-support-darunavir-based-hiv-treatments-for-coronavirus

26. Miller DK, Lenard J. Antihistaminics, local anesthetics, and other amines as antiviral agents. PNAS. 1981 Jun 1;78(6):3605–9.

27. Keyaerts E, Vijgen L, Maes P, Neyts J, Van Ranst M. In vitro inhibition of severe acute respiratory syndrome coronavirus by chloroquine. Biochem Biophys Res Commun. 2004 Oct 8;323(1):264–8.

28. Paton NI, Lee L, Xu Y, Ooi EE, Cheung YB, Archuleta S, et al. Chloroquine for influenza prevention: a randomised, double-blind, placebo controlled trial. Lancet Infect Dis. 2011 Sep;11(9):677–83.

29. Tricou V, Minh NN, Van TP, Lee SJ, Farrar J, Wills B, et al. A Randomized Controlled Trial of Chloroquine for the Treatment of Dengue in Vietnamese Adults. PLOS Neglected Tropical Diseases. 2010 Aug 10;4(8):e785.

30. Roques P, Thiberville S-D, Dupuis-Maguiraga L, Lum F-M, Labadie K, Martinon F, et al. Paradoxical Effect of Chloroquine Treatment in Enhancing Chikungunya Virus Infection. Viruses. 2018 17;10(5).

31. Wang M, Cao R, Zhang L, Yang X, Liu J, Xu M, et al. Remdesivir and chloroquine effectively inhibit the recently emerged novel coronavirus (2019-nCoV) in vitro. Cell Res. 2020 Mar;30(3):269–71.

32. Gao J, Tian Z, Yang X. Breakthrough: Chloroquine phosphate has shown apparent efficacy in treatment of COVID-19 associated pneumonia in clinical studies. Biosci Trends. 2020 Mar 16;14(1):72–3.

33. Cortegiani A, Ingoglia G, Ippolito M, Giarratano A, Einav S. A systematic review on the efficacy and safety of chloroquine for the treatment of COVID-19. J Crit Care. 2020;57:279–83.

34. multicenter collaboration group of Department of Science and Technology of Guangdong Province and Health Commission of Guangdong Province for chloroquine in the treatment of novel coronavirus pneumonia. [Expert consensus on chloroquine phosphate for the treatment of novel coronavirus pneumonia]. Zhonghua Jie He He Hu Xi Za Zhi. 2020 Mar 12;43(3):185–8.

35. Touret F, de Lamballerie X. Of chloroquine and COVID-19. Antiviral Res. 2020 Mar 5;177:104762.

36. Colson P, Rolain J-M, Lagier J-C, Brouqui P, Raoult D. Chloroquine and hydroxychloroquine as available weapons to fight COVID-19. Int J Antimicrob Agents. 2020 Mar 4;105932.

37. Yao X, Ye F, Zhang M, Cui C, Huang B, Niu P, et al. In Vitro Antiviral Activity and Projection of Optimized Dosing Design of Hydroxychloroquine for the Treatment of Severe Acute Respiratory Syndrome Coronavirus 2 (SARS-CoV-2). Clin Infect Dis. 2020 Mar 9;

38. Harmon B, Campbell N, Ratner L. Role of Abl kinase and the Wave2 signaling complex in HIV-1 entry at a post-hemifusion step. PLoS Pathog. 2010 Jun 17;6(6):e1000956.

39. Newsome TP, Weisswange I, Frischknecht F, Way M. Abl collaborates with Src family kinases to stimulate actin-based motility of vaccinia virus. Cell Microbiol. 2006 Feb;8(2):233–41.

40. Coyne CB, Bergelson JM. Virus-induced Abl and Fyn kinase signals permit coxsackievirus entry through epithelial tight junctions. Cell. 2006 Jan 13;124(1):119–31.

41. García M, Cooper A, Shi W, Bornmann W, Carrion R, Kalman D, et al. Productive Replication of Ebola Virus Is Regulated by the c-Abl1 Tyrosine Kinase. Sci Transl Med. 2012 Feb 29;4(123):123ra24.

42. Kouznetsova J, Sun W, Martínez-Romero C, Tawa G, Shinn P, Chen CZ, et al. Identification of 53 compounds that block Ebola virus-like particle entry via a repurposing screen of approved drugs. Emerg Microbes Infect. 2014 Dec;3(12):e84.

43. Sisk JM, Frieman MB, Machamer CE. Coronavirus S protein-induced fusion is blocked prior to hemifusion by Abl kinase inhibitors. J Gen Virol. 2018;99(5):619–30.

44. Dyall J, Coleman CM, Hart BJ, Venkataraman T, Holbrook MR, Kindrachuk J, et al. Repurposing of clinically developed drugs for treatment of Middle East respiratory syndrome coronavirus infection. Antimicrob Agents Chemother. 2014 Aug;58(8):4885–93.

45. Coleman CM, Sisk JM, Mingo RM, Nelson EA, White JM, Frieman MB. Abelson Kinase Inhibitors Are Potent Inhibitors of Severe Acute Respiratory Syndrome Coronavirus and Middle East Respiratory Syndrome Coronavirus Fusion. J Virol. 2016 01;90(19):8924–33.

46. Caldemeyer L, Dugan M, Edwards J, Akard L. Long-Term Side Effects of Tyrosine Kinase Inhibitors in Chronic Myeloid Leukemia. Curr Hematol Malig Rep. 2016 Apr;11(2):71–9.

47. Crugnola M, Castagnetti F, Breccia M, Ferrero D, Trawinska MM, Abruzzese E, et al. Outcome of very elderly chronic myeloid leukaemia patients treated with imatinib frontline. Ann Hematol. 2019 Oct;98(10):2329–38.

48. Horne GA, Stobo J, Kelly C, Mukhopadhyay A, Latif AL, Dixon-Hughes J, et al. A randomised phase II trial of hydroxychloroquine and imatinib versus imatinib alone for patients with chronic myeloid leukaemia in major cytogenetic response with residual disease. Leukemia. 2020 Jan 10;

49. Furuta Y, Gowen BB, Takahashi K, Shiraki K, Smee DF, Barnard DL. Favipiravir (T-705), a novel viral RNA polymerase inhibitor. Antiviral Res. 2013 Nov;100(2):446–54.

50. Furuta Y, Komeno T, Nakamura T. Favipiravir (T-705), a broad spectrum inhibitor of viral RNA polymerase. Proc Jpn Acad, Ser B, Phys Biol Sci. 2017;93(7):449–63.

51. Sissoko D, Laouenan C, Folkesson E, M'Lebing A-B, Beavogui A-H, Baize S, et al. Experimental Treatment with Favipiravir for Ebola Virus Disease (the JIKI Trial): A Historically Controlled, Single-Arm Proof-of-Concept Trial in Guinea. PLoS Med. 2016 Mar;13(3):e1001967.

52. Nguyen THT, Guedj J, Anglaret X, Laouénan C, Madelain V, Taburet A-M, et al. Favipiravir pharmacokinetics in Ebola-Infected patients of the JIKI trial reveals concentrations lower than targeted. PLoS Negl Trop Dis. 2017 Feb;11(2):e0005389.

53. Dong L, Hu S, Gao J. Discovering drugs to treat coronavirus disease 2019 (COVID-19). Drug Discov Ther. 2020;14(1):58–60.

54. Madelain V, Mentré F, Baize S, Anglaret X, Laouénan C, Oestereich L, et al. Modeling Favipiravir Antiviral Efficacy Against Emerging Viruses: From Animal Studies to Clinical Trials. CPT Pharmacometrics Syst Pharmacol. 2020 May;9(5):258–71.

55. Lu R, Zhao X, Li J, Niu P, Yang B, Wu H, et al. Genomic characterisation and epidemiology of 2019 novel coronavirus: implications for virus origins and receptor binding. The Lancet [Internet]. 2020 Jan 30 [cited 2020 Jan 30];0(0). Available from: https://www.thelancet.com/journals/lancet/article/PIIS0140-6736(20)30251-8/abstract

56. Santos RAS, Simoes e Silva AC, Maric C, Silva DMR, Machado RP, de Buhr I, et al. Angiotensin-(1-7) is an endogenous ligand for the G protein-coupled receptor Mas. Proc Natl Acad Sci USA. 2003 Jul 8;100(14):8258–63.

57. Ferrario CM, Jessup J, Chappell MC, Averill DB, Brosnihan KB, Tallant EA, et al. Effect of angiotensin-converting enzyme inhibition and angiotensin II receptor blockers on cardiac angiotensin-converting enzyme 2. Circulation. 2005 May 24;111(20):2605–10.

58. Imai Y, Kuba K, Rao S, Huan Y, Guo F, Guan B, et al. Angiotensin-converting enzyme 2 protects from severe acute lung failure. Nature. 2005 Jul 7;436(7047):112–6.

59. Kuba K, Imai Y, Rao S, Gao H, Guo F, Guan B, et al. A crucial role of angiotensin converting enzyme 2 (ACE2) in SARS coronavirus-induced lung injury. Nat Med. 2005 Aug;11(8):875–9.

60. Liu Y, Huang F, Xu J, Yang P, Qin Y, Cao M, et al. Anti-hypertensive Angiotensin II receptor blockers associated to mitigation of disease severity in elderly COVID-19 patients. medRxiv. 2020 Mar 27;2020.03.20.20039586.

61. Deppe S, Böger RH, Weiss J, Benndorf RA. Telmisartan: a review of its pharmacodynamic and pharmacokinetic properties. Expert Opin Drug Metab Toxicol. 2010 Jul;6(7):863–71.

62. Group TPIW. A Randomized, Controlled Trial of ZMapp for Ebola Virus Infection. New England Journal of Medicine. 2016 Oct 13;375(15):1448–56.

63. Duvignaud A, Jaspard M, Etafo IC, Serra B, Abejegah C, Gabillard D, et al. Lassa fever clinical course and setting a standard of care for future randomized trials: A protocol for a cohort study of Lassa-infected patients in Nigeria (LASCOPE). Travel Medicine and Infectious Disease. 2020 Jan 21;101557.

64. TEMPRANO ANRS 12136 Study Group, Danel C, Moh R, Gabillard D, Badje A, Le Carrou J, et al. A Trial of Early Antiretrovirals and Isoniazid Preventive Therapy in Africa. N Engl J Med. 2015 Aug 27;373(9):808–22.

65. Royston P, Barthel FM-S, Parmar MK, Choodari-Oskooei B, Isham V. Designs for clinical trials with time-to-event outcomes based on stopping guidelines for lack of benefit. Trials. 2011 Mar 18;12:81.

66. Bratton DJ, Phillips PPJ, Parmar MKB. A multi-arm multi-stage clinical trial design for binary outcomes with application to tuberculosis. BMC Med Res Methodol. 2013 Nov 14;13:139.

67. O'Brien PC, Fleming TR. A multiple testing procedure for clinical trials. Biometrics. 1979 Sep;35(3):549–56.

68. Lan KKG, DeMets DL. Discrete Sequential Boundaries for Clinical Trials. Biometrika. 1983;70(3):659–63.

69. Huang C, Wang Y, Li X, Ren L, Zhao J, Hu Y, et al. Clinical features of patients infected with 2019 novel coronavirus in Wuhan, China. Lancet. 2020 Feb 15;395(10223):497–506.

70. Liu WJ, Zhao M, Liu K, Xu K, Wong G, Tan W, et al. T-cell immunity of SARS-CoV: Implications for vaccine development against MERS-CoV. Antiviral Res. 2017 Jan;137:82–92.

71. Shakoory B, Carcillo JA, Chatham WW, Amdur RL, Zhao H, Dinarello CA, et al. Interleukin-1 Receptor Blockade Is Associated With Reduced Mortality in Sepsis Patients With Features of Macrophage Activation Syndrome: Reanalysis of a Prior Phase III Trial. Crit Care Med. 2016 Feb;44(2):275–81.

72. Lhomme E, Hejblum BP, Lacabaratz C, Wiedemann A, Lelièvre J-D, Levy Y, et al. Analyzing cellular immunogenicity in vaccine clinical trials: a new statistical method including non-specific responses for accurate estimation of vaccine effect. J Immunol Methods. 2020 Feb;477:112711.

73. Lévy Y, Thiébaut R, Montes M, Lacabaratz C, Sloan L, King B, et al. Dendritic cell-based therapeutic vaccine elicits polyfunctional HIV-specific T-cell immunity associated with control of viral load. Eur J Immunol. 2014 Sep;44(9):2802–10.

74. Vittinghoff E, McCulloch CE. Relaxing the rule of ten events per variable in logistic and Cox regression. Am J Epidemiol. 2007 Mar 15;165(6):710–8.

75. Agniel D, Hejblum BP. Variance component score test for time-course gene set analysis of longitudinal RNA-seq data. Biostatistics. 2017 Oct 1;18(4):589–604.

76. Thabane L, Ma J, Chu R, Cheng J, Ismaila A, Rios LP, et al. A tutorial on pilot studies: the what, why and how. BMC Medical Research Methodology. 2010 Jan 6;10(1):1.
