# Supplementary material for: Home Treatment of Older People with Symptomatic SARS-CoV-2 Infection (COVID-19): A structured Summary of a Study Protocol for a Multi-Arm Multi-Stage (MAMS) Randomized Trial to Evaluate the Efficacy and Tolerability of Several Experimental Treatments to Reduce the Risk of Hospitalisation or Death in outpatients aged 65 years or older (COVERAGE trial)
Source: Trials. 2020 Oct 13;21:846. doi: 10.1186/s13063-020-04619-1 (PMC7552584; doi:10.1186/s13063-020-04619-1)
Supplement: Supplementary file 3 — Additional file 3. List of members of the COVERAGE study group. [file 13063_2020_4619_MOESM3_ESM.pdf]

COVERAGE Study Group

Version 1.0 – July 4th, 2020

- ***Scientific Advisory Board (SAB):***

Voting members*:* X. de Lamballerie (Chair), X. Anglaret, L. Atlani-Duault, F. Chauvin, T. Darnaud, P-L. Druais, V. Dubee, J. Dupouy, A. Gimbert, J-P. Joseph, V. Journot, D. Lebeaux, B. Lefèvre, E. Lhomme, D. Malvy (Coordinating investigator), F. Mentré, J-F. Michel, J-L. Montastruc, J. Orne Gliemann, L. Piroth, L. Richert, C. Roussillon, R. Thiebaut, L. Wittkop

Non-voting members*:* S. Bouchet, C. Cazanave, S. Conde, A. Cremer, S. Djabarouti, A. Duvignaud, C. Gil Jardine, L. Hardel, M-E. Lafon, L. Letinier, S. Marchi, L. Moinot, D. Nguyen, R. Onaisi, I. Pellegrin, T. Pistone, T. Schaeverbeke

- ***Data Safety Monitoring Board (DSMB):*** D. Costagliola (Chair), E. Bellissant, G. Gavazzi, A-M. Taburet, P. Tattevin, S. Walker

- ***Coordinating Unit***

CIC-EC 1401/EUCLID: L. Richert (head), S. Canete, N. Chaghil, S. Daoui, G. Dupouy, L. Esterle, V. Favreau, A. Gelley, S. Gillet, L. Hardel, M. Kanté, E. Lhomme, S. Martiren, L. Moinot, L. Pinoges, E. Rouch, C. Schwimmer, R. Sitta, M. Termote, C. Wallet, L. Wittkop

MEREVA: X. Anglaret (head), E. Balestre, A. Beuscart, C. Bonnier, C. Cazes, C. Chazallon, G. Clouet, M. Daures, D. Gabillard, G. Habiyambere, V. Journot, L. Lambert, M. Loniewski, S. Karcher, J. Le Carrou, O. Marcy, V. Murat, J. Orne Gliemann, M. Plazy

- ***CHU Bordeaux, trial sponsor:*** J. Belcastro (head) , P. Beaufrère, T. Brice, P. Cassaï, A. Gimbert, S. Marchi, V. Marty, F. Nacka, P. Poulizac, S. Regueme, M. Rousset, C. Roussillon, F. Salvo, S. Vautrat

- ***Coordinating Pharmacy:*** S. Djabarouti (head), B. Ghezzoul, M. Gigan, J. Le Chanjour, C. Langlade, P. Mora, B. Sourisseau

- ***Laboratory:***

Bordeaux: I. Pellegrin (head), S. Bouchet, C. Cognet, I. Garrigue, J. Jeanpetit, M-E. Lafon, A. Pouzet, A. Tarricone, P. Trimoulet, A. Voldoire

Créteil (sub-study): C. Lacabaratz (head), H. Hocini

- ***Bordeaux study site:***

Investigating physicians: D. Malvy (principal investigator), C. Bez, E. Collomb, C. Dubourdieu, A. Duvignaud, M. Galinski, P. Gibaud, C. Gil-Jardine, J. Guillot-Warin, C. Lebouc, A. Leger, V. Lengline, C. Loizeau, M. Mayenc, D. Nguyen, M. Odorico, R. Onaisi, T. Pistone, F. Sacher, J. Scandella

Coordination: X. Anglaret (head), R. Brégéras, N. Brionnec, S. Condé, C. Gazille, C. Grenier, G Kouame, J-B. N'Takpe, Z. Pascual

Other contributors for Bordeaux study site:

Isabelle GOASDOUE	Nurse	Medical mobile team
Céline DUPUIS	Nurse	Medical mobile team
Samra MUSIC	Nurse	Medical mobile team
Christel GUEMISE	Nurse	Medical mobile team
Sabine VALETTE	Nurse	Medical mobile team
Laurence FRICHOT	Nurse	Medical mobile team
Ornella KOMBOLI	Nurse	Medical mobile team
Lucas GRODECOEUR	Nurse	Medical mobile team
Théo DELBOS	Nurse	Medical mobile team
Antoine MARCHAND	Nurse	Medical mobile team
Jean-Baptiste CANDONI	Nurse	Medical mobile team
Sarai QUINTELA SALAS	Nurse	Medical mobile team
Anne-Laure BARIS	Nurse	Medical mobile team
Aurélie TRAORE	Nurse	Medical mobile team
Carine MOUTOUSSAMY	Nurse	Medical mobile team
Marie-Hélène FALCOU	Nurse	Medical mobile team
Marie-Charlotte CALLU	Nurse	Medical mobile team
Anna GAUTIER	Nurse	Medical mobile team
Marion LE BELICARD	Nurse	Medical mobile team
Sandra TRESCASES	Nurse	Medical mobile team
Marcia Sofia DIAS COSA	Nurse	Medical mobile team
Cécile LAFITTE-TROUQUE	Nurse	Medical mobile team
Charline RAMOS	Nurse	Medical mobile team
Nicolas VOISIN	Nurse	Medical mobile team
Cléa SICCARDI	Nurse	Medical mobile team
Mathieu ADIGO	Nurse	Medical mobile team
Gaëlle GUESDON	Nurse	Medical mobile team
Audrey JUINIER	Nurse	Medical mobile team
François NIEGER	Nurse	Medical mobile team
Romain GEYNES	Nurse	Medical mobile team
Victor SALVAT	Nurse	Medical mobile team
Audrey COUZI	Nurse	Medical mobile team
Alan CAER	Nurse	Medical mobile team
Pierre-Marie BONTEMPS	Nurse	Medical mobile team
Cécile MONGABURE	Nurse	Medical mobile team

Quentin BECHADE	Nurse	Medical mobile team
Chloé LE GLATIN	Nurse	Medical mobile team
Pierre JOUANLAU	Nurse	Medical mobile team
Isabelle BERNIS	Nurse	Medical mobile team
Alice LEREVEREND	Nurse	Medical mobile team
Alice HERTEAU	Medical resident	Ambulatory testing centre
Fanny VELARDO	Medical resident	Ambulatory testing centre
Aurore GONTHIER	Medical resident	Ambulatory testing centre
Jules DUBOIS	Medical resident	Ambulatory testing centre
Aydin SANAVI	Medical resident	Ambulatory testing centre
Quentin LHUAIRE	Medical resident	Ambulatory testing centre
Sara VERHAGHE	Medical resident	Ambulatory testing centre
Léa RAGOT	Medical resident	Ambulatory testing centre
Roxane BAILLEUL	Medical resident	Ambulatory testing centre
Melaine DUPUY	Medical resident	Ambulatory testing centre
Adrien AUBER	Medical resident	Ambulatory testing centre
Deborah VILLANI	Medical resident	Ambulatory testing centre
Liliane MOUCHE	Medical resident	Ambulatory testing centre
Fifamè GBAGONOU	Physician	Partnership office
Marion DESTENAY	Medical resident	Partnership office
Hugo LE BRETON	Medical resident	Partnership office
Elisabeth VINCENT	Medical resident	Partnership office
Brianne BREGER	Medical resident	Partnership office
Carlotta CANDONI	Medical resident	Partnership office
Kévin OUAZZANI	Medical resident	Partnership office
Mathilde PUGES	Physician	COVID-19 call centre
Mathilde CARRER	Medical resident	COVID-19 call centre
Laure BARTHOD	Medical resident	COVID-19 call centre
Claire TIVENEZ	Medical resident	COVID-19 call centre
Yannis BELAROUSSI	Medical resident	COVID-19 call centre
Aurélie CLAUSSE	Medical resident	COVERAGE trial medical regulation
Anne-Sophie EZANNO	Medical resident	COVERAGE trial medical regulation
Côme LOURDE	Medical resident	COVERAGE trial medical regulation
Gaël GALLI	Medical resident	COVERAGE trial medical regulation
Tara FLEUR	Medical Biology Resident	COVERAGE biological regulation
Pauline RATUIT	Medical Biology Resident	COVERAGE biological regulation
Clément CHEMIN	Medical Biology Resident	COVERAGE biological regulation

Camille MORIVAL	Pharmacist	Trial drugs’ supply
Pauline ETIENNE	Pharmacy resident	Trial drugs’ supply
Sacha BODIN	Pharmacy resident	Trial drugs’ supply
Damien LE LEU	Pharmacy resident	Trial drugs’ supply
Lauriane CORNUAULT	Pharmacy resident	Trial drugs’ supply
Victor STANISLAS	Pharmacy resident	Trial drugs’ supply
Clémence HUYNH	Pharmacy resident	Trial drugs’ supply
Sarah MONIC	Pharmacy resident	Trial drugs’ supply
Théo BAZIN	Pharmacy student	Trial drugs’ supply
Julie PERROT	Pharmacy student	Trial drugs’ supply
Alexandre THEVENIAULT	Pharmacy student	Trial drugs’ supply
Dominique SAN MIGUEL	Pharmacy student	Trial drugs’ supply
Eva MEUNIER	Pharmacy student	Trial drugs’ supply
Jean-Baptiste MONCE	Pharmacy student	Trial drugs’ supply
Camille ETCHEVERRIA	Pharmacy student	Trial drugs’ supply
Anthony MARQUES	Pharmacy student	Trial drugs’ supply
Basile DEJONGHE	Medical resident	Logistical support
David PECAK	Medical resident	Logistical support
Camille DE POUS	Medical resident	Logistical support
Nguyen VAN THIEN	Medical resident	Logistical support
Caroline PUYO	Medical student	Logistical support
Joffrey CALLEGARIN	Medical student	Logistical support
Camille GUYON	Pharmacy student	Logistical support
Yasmine BENDANI	Medical student	Logistical support
Carla TACHOT	Pharmacy student	Logistical support
Elorri LARREGARAY	Biological & health sciences student	Logistical support
Capucine MESNARD	Medical student	Medical mobile team support
Vincent PASETTI	Medical student	Medical mobile team support
Chloé LACARRIERE	Medical student	Medical mobile team support
Nadia RENTCHLER	Medical student	Medical mobile team support
Richard NGUYEN	Medical student	Medical mobile team support
Camille LAFON	Medical student	Medical mobile team support
Gaétan BASILE	Medical student	Medical mobile team support
Elisa MERCIER	Medical student	Medical mobile team support
Yves TRUPIN	Medical student	Medical mobile team support
Clara BODEREAU	Medical student	Medical mobile team support
Fany NOUGUE-DESSUS	Medical student	Medical mobile team support
Alexis DUCOS	Medical student	Medical mobile team support
Sophie NABAIS	Medical student	Medical mobile team support
Max DADJO	Medical student	Medical mobile team support
Arthur GIRODEAU	Medical student	Medical mobile team support

Nyere GIBSON	Medical student	Administrative support
Léon RAYNAUD	Medical student	Administrative support
Maika PATOUILLE	Medical student	Administrative support
Emma DIALLO	Medical student	Administrative support
Juliette PETIT	Medical student	Administrative support
Marcus PALU	Medical student	Administrative support
Lorena SANCHEZ-BLANCO	Clinical Research Assistant	Rythmology platform

- ***Dijon Study site***: L. Piroth (principal investigator), C. Binquet, M. Bouctot, A. Lamotte Felin, C. Simonel

- ***Nancy Study site:*** B. Lefevre (principal investigator), C. Daguin, E. Dauchy, M. Gilg, P. Rossignol

- ***Toulouse Study site***: J. Dupouy (principal investigator),
  - **Coordination:** P. Gauteul, J. Germain, C. Lebely,
  - **Administrative coordination:** F. Gross
  - **Clinical Research Assistant:** C. Rivière, S. Bras
  - **General Practice coordination:** S. Oustric, T. Brillac, J.C. Poutrain, L. Gimenez
  - **CIC medical doctors:** O. Rascol, C. Thalamas, M. Galitsky, F. Calvas, H. Catala, M. Lapeyre-Mestre, A. Sommet
  - **CIC nurses:** S. Rolland, S. Bonnet, B. Lagarde
  - **Hospital pharmacists:** F. Eyvrard, C. Sorli
  - **Partnership office:** P. Boyer, C. Landon, B. Ortala, A. Gervais, M. Courderc, J.C. Poutrain, E. Escourrou, M.E. Rougé-Bugat, F. Marchadier, S. Mathé, A. Gruber, A. Boucault, S.H. Eveno

- ***Paris-HEGP Study site***: D. Lebeaux (principal investigator), J. Djadi-Prat, A-I Tropeano

- ***Corse Study site***:
    - ***Investigating physicians***: T. Darnaud (principal investigator), C. Hébert, P. Casta, C. Costa, M. Bonnard, S. Guerrini
    - ***Coordination*** : I. Giusti, S. Provent, D. Poitrenaud, J. Besseron, D. Zamponi, X. Pieri, A. Grisoni, MH. Pietri-Zani
    - **Other contributors for Corse study site:**

Pauline CASTA	Physician	Medical mobile team
Christophe HEBERT	Physician	Medical mobile team
Cécilia COSTA	Physician	Medical mobile team
Marion BONNARD	Medical resident	Medical mobile team
Serena GUERRINI	Medical resident	Medical mobile team
Julie BESSERON	Pharmacist	Trial Drug's supply
Sandra SALINI	Pharmacist	Trial Drug's supply
Benjamin DARMON	Pharmacist	Trial Drug's supply
Dominique BERTEI	Medical biology	Biological regulation
Hélène DOLFI FIETTE	Medical biology	Biological regulation

- ***Luxembourg Study site***: J-F. Michel (principal investigator), V. Arendt, J. Cimino, M. Gantenbein, M. Vaillant

- ***Paris VII Study site:*** J. Le Bel (principal investigator), A. Aim-Eusebi, C. Flipo, L. Rossignol

- ***Others contributors:*** T. Alcouffe, V. Briand, S. Coudray, D. Charles, H. Jacquet, C. Levy-Marchal, J. Raude, P. Vellozzo, P. Whitheway, Nathan Peiffer-Smadja
